# MR-PheWAS: hypothesis prioritization among potential causal effects of body mass index on many outcomes, using Mendelian randomization

**DOI:** 10.1038/srep16645

**Published:** 2015-11-16

**Authors:** Louise A. C. Millard, Neil M. Davies, Nic J. Timpson, Kate Tilling, Peter A. Flach, George Davey Smith

**Affiliations:** 1 MRC Integrative Epidemiology Unit (IEU) at the University of Bristol, University of Bristol, Bristol; 2 Intelligent Systems Laboratory, Department of Computer Science, University of Bristol, UK

## Abstract

Observational cohort studies can provide rich datasets with a diverse range of phenotypic variables. However, hypothesis-driven epidemiological analyses by definition only test particular hypotheses chosen by researchers. Furthermore, observational analyses may not provide robust evidence of causality, as they are susceptible to confounding, reverse causation and measurement error. Using body mass index (BMI) as an exemplar, we demonstrate a novel extension to the phenome-wide association study (pheWAS) approach, using automated screening with genotypic instruments to screen for causal associations amongst any number of phenotypic outcomes. We used a sample of 8,121 children from the ALSPAC dataset, and tested the linear association of a BMI-associated allele score with 172 phenotypic outcomes (with variable sample sizes). We also performed an instrumental variable analysis to estimate the causal effect of BMI on each phenotype. We found 21 of the 172 outcomes were associated with the allele score at an unadjusted p < 0.05 threshold, and use Bonferroni corrections, permutation testing and estimates of the false discovery rate to consider the strength of results given the number of tests performed. The most strongly associated outcomes included leptin, lipid profile, and blood pressure. We also found novel evidence of effects of BMI on a global self-worth score.

Epidemiology is typically hypothesis-driven, using prior knowledge to specify a hypothesis to be tested. However this can bias epidemiological research to hypotheses where there is a prior belief that an association exists. Also, the analyst’s research interests and preconceptions about the composition of causal pathways affects the hypotheses they decide to test. Candidate gene studies in genetic epidemiology provide an example of this, with hypothesis-driven approaches producing largely non-replicable findings^1^,^2^. An alternative approach is to use hypothesis-searching methods to identify associations to follow-up, where a set of hypotheses is systematically tested using a pre-specified test for association. For example, the hypothesis-searching genome-wide association study (GWAS) approach, with appropriate statistical testing, has generated highly replicable results^3^,^4^. There is little reason to presume that hypothesis-driven phenotypic studies will be substantially more successful than candidate gene studies, as their reporting in the literature is also likely to be biased due to small sample sizes and publication bias, in addition to the widely appreciated problems inherent in observational epidemiological research^5^.

Epidemiologists have struggled to identify causal relationships using observational data because an observed association between an exposure and outcome may be due not only to chance but also to unknown/unmeasured confounders, residual confounding from measurement error in known confounders, and reverse causation^6^,^7^. Mendelian randomization can help researchers infer causation by using instrumental variables (IVs) constructed from genetic variants^8^,^9^. This follows from the two (approximate) laws of Mendelian genetics: the Law of Segregation (Mendel’s first law) and the Law of Independent Assortment (Mendel’s second law). These laws imply that, linkage disequilibrium accepted, genetic variants are unlikely to be associated with confounding phenotypic or genetic factors^10^.

A robust approach to Mendelian randomization is to estimate the association of genetic variants directly with the outcome. This provides a valid test of whether an exposure causes an outcome and only depends on the three core instrumental variable assumptions^11^. These are: (1) the instrumental variable is associated with the exposure, (2) the instrumental variable is not associated with the factors that confound the association between the exposure and outcome, and (3) the instrumental variable is associated with the outcome solely through the exposure^12^. Pleiotropy, linkage disequilibrium or population stratification can invalidate these assumptions. In order to estimate the size of the effect of an exposure on an outcome, the exposure phenotype must also be used in the analysis. When the exposure is used in the analysis the instrumental variable assumptions may be invalidated in other ways, such as if the instrument affects the outcome through the exposure phenotype at other time points than those included in the analysis. For instance, if body mass index (BMI) at age 2 and at age 25 both affected coronary heart disease, the published allele score for BMI could not identify the independent effects of BMI at these time points^13^. Furthermore, researchers must impose stronger, point identifying assumptions to estimate the size of the effect of the exposure on the outcome. For example, epidemiologists have commonly assumed constant treatment effects or no effect modification for continuous outcomes, or no effect modification for binary outcomes^14^. Researchers can investigate the validity of the core instrumental variable assumptions if multiple genetic variants are associated with the exposure. If two or more variants affect an exposure through different causal pathways, and the core instrumental variable assumptions and either of the point identifying assumptions hold, the variants should estimate the same size of causal effect of the exposure on the outcome^12^.

Hypothesis-searching is an established method to find genomic associations in GWAS, where little may be known about which genes influence an outcome. Recently, there have also been environment-wide association studies (EWAS)^15^,^16^ that search a large set of potential hypotheses (such as the association of blood pressure with a range of nutrients^16^). One study^17^ used previous GWAS and EWAS results (from^15^) to inform a hypothesis space to search for gene-environment interactions in order to minimize the number of tests carried out (which grows exponentially for interaction tests). The phenome-wide association study (pheWAS) approach has been used to investigate the association between a set of genetic variants and a set of phenotypes, testing the association of each genetic variant, phenotype pair individually^18^. The rapidly expanding number of genetic variants with known associations means the pheWAS approach can be extended to test for causal relationships using Mendelian randomisation^15^,^19^,^20^.

We aimed to illustrate how Mendelian randomization can be used to investigate the effects of an exposure on a large set of outcomes, to identify outcomes potentially causally influenced by the exposure. We call this approach MR-pheWAS (Mendelian randomisation-pheWAS). As with GWAS, this is a screening approach where identified associations need to be validated through targeted replication studies. The aim of this work is to illustrate this hypothesis-searching method, and we use a set of positive controls to evaluate its effectiveness.

We will use the relationship between BMI, BMI genetic variants and a range of outcomes as an exemplar. BMI has a close relationship with many traits and is associated with diseases such as type 2 diabetes and cardiovascular disease^21^. An association between BMI and a phenotype may be due to confounding or because the phenotype affects BMI, rather than because BMI affects the phenotype. For instance, clinical trials have shown BMI is affected by environmental factors such as diet and exercise^22^,^23^. We build upon previous Mendelian randomization studies that have investigated the causal effects of BMI on inflammation, cancer, age at menarche, diabetes, atherosclerosis risk and blood pressure and hypertension^21^,^24^,^25^,^26^,^27^,^28^,^29^,^30^,^31^,^32^,^33^ and bi-directional studies that have analysed the effects of BMI and a second exposure such as C-reactive protein^28^,^34^, serum uric acid^35^,^36^, vitamin-D^37^ and fetuin-A^38^.

## Results

The association between the BMI allele score and observed BMI across childhood strengthens with age and stabilizes at around age 10 (Table 1). A standard deviation increase in BMI allele score was associated with a 0.163 standard deviation increase in log BMI at age 8 (95% confidence interval (CI): 0.14, 0.19, F = 140.66). Furthermore, we found little evidence of associations with common socio-economic confounders compared with many strong associations for observational BMI at age 8 (Table 2). These tests support the notion that the BMI allele score may be a valid instrument for life-long BMI.

**Figure 1:**
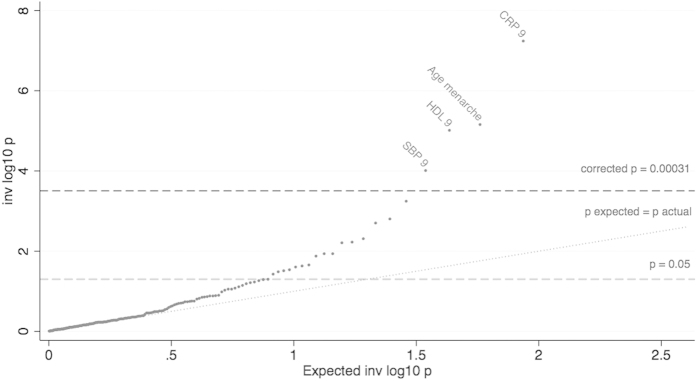
QQ-plot of the associations between the BMI allelic score and the 172 outcomes. Association of log BMI age 8 with outcomes, of the stage 1 tests. Using the original dataset with variable number of individuals for each outcome. Tests performed with the Stata regress command and robust option. Top result leptin is not shown as P value too small. Corrected P = 0.00023 line: The Bonferroni corrected P = 0.05, accounting for the 160 tests (excluding validation set) performed. P expected = actual line: The expected trajectory, assuming the P values are uniformly distributed.

**Figure 2:**
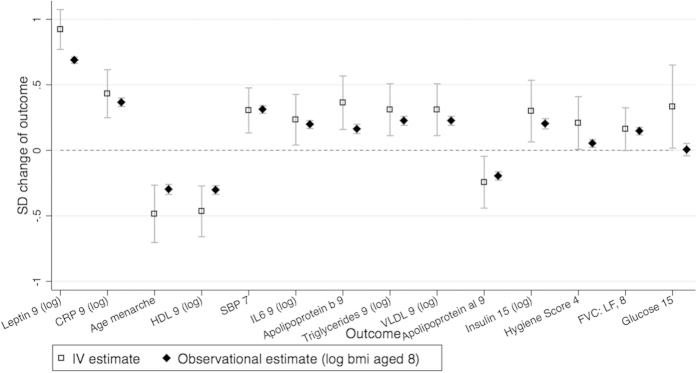
A comparison of the observational and instrumental variable estimates for continuous outcomes. The standard deviation change of outcome for a 1 SD increase of log BMI aged 8. IV estimate of effect using two-stage least squares regression of log BMI at age 8 as the exposure, with robust option. Observational estimates are the SD change of the outcome for a 1 SD increase in log BMI at age 8. Graphical illustration of the results in Table 4.

**Figure 3:**
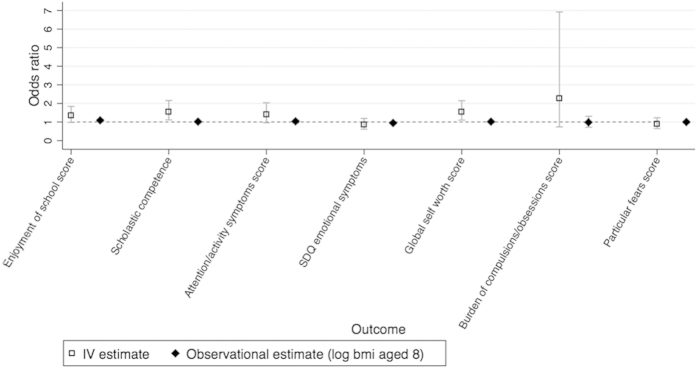
A comparison of the observational and instrumental variable estimates for binary outcomes. Odds ratio between groups of outcomes, for a 1 SD change of log BMI aged 8. IV estimate of effect using two-stage least squares regression of log BMI at age 8 as the exposure, with robust option. Observational estimates are the odds ratio between outcome groups. Graphical illustration of the results in Table 4. Categories for binary variables given in Supplementary Table 7.

Our stage 1 tests found the BMI allele score was associated with 21 outcomes, using an unadjusted P < 0.05 threshold (Table 3). Of these, 14 outcomes were from the 160 outcomes we randomly included in our dataset (test of proportions P = 0.030), compared to 8 expected by chance alone (160 × 0.05, making the conservative assumption that all outcomes are uncorrelated). Hence we would expect 6 of the 14 identified outcomes to be true associations (false discovery rate of 0.571). We found stronger associations than would be expected by chance, illustrated by the QQ plot in Fig. 1, suggesting that BMI affects many outcomes. After Bonferroni correction only HDL at age 9 was found with a P value below P < 0.05 whereas using the permutation P values we found 8 associations with P <0.05. In comparison, we found 57 stage 1 associations with P < 0.05 using observational BMI at age 8. Of these, 48 were from the 160 randomly included in our dataset (test of proportions P = <0.001), compared to 8 expected by chance alone. The instrumental variable effect estimates (stage 2 results) are given in Table 4 and Figs 2 and 3 (and observational estimates are also provided for comparison).

The stage 1 direct tests identified several known associations, such as with leptin and age at menarche. The two-stage least squares IV analysis estimated that a 1 SD increase in log BMI at age 8 increased log leptin at age 9 by 0.92 SD (95% confidence interval (CI): 0.77, 1.07), which equates to a 0.05% increase in leptin per 1% increase in BMI age 8. A 1 SD increase in log BMI at age 8 was associated with a 201.7 day earlier age at menarche (95% CI: 112.3, 291.1), which equates to a 15.5 day earlier age at menarche (95% CI: 8.53, 22.57) for a 1% increase in BMI age 8. We also identified novel effects of BMI. For instance, a 1 SD increase in log BMI at age 8 increased the odds of having a global self-worth score ≥20 by 53.7% (95% CI: 10.2, 214.2), which equates to a 37.1% (95% CI: 7.4, 75.1) increase in the odds of having a global self-worth score ≥20, for a 10% increase in BMI age 8. We list all outcomes in our dataset in Supplementary Table 3 and the results of the stage 1 tests in Supplementary Table 4, so that readers can view results where the CI includes the null value. The observational estimates were nearer the null than the IV estimates, and we found differences between the IV and observational estimates for 6 phenotypes, using the Durbin-Wu-Hausman test (Table 4).

### Evidence of violation of IV assumptions

**Figure 4:**
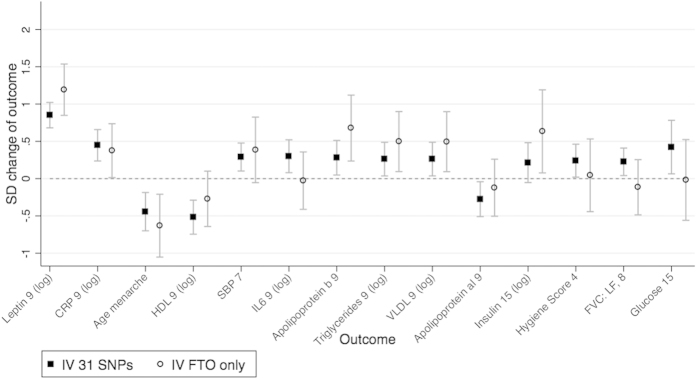
Testing invalidity of IV assumptions: associations of two instrumental variables using distinct SNP subsets, for continuous outcomes. Comparison between the SNP subsets: (1) 31 SNPs (excluding FTO SNP) and (2) the FTO SNP only. IV estimate of effect using two-stage least squared regression of log BMI at age 8 as the exposure. Graphical illustration of the results in Table 5.

**Figure 5:**
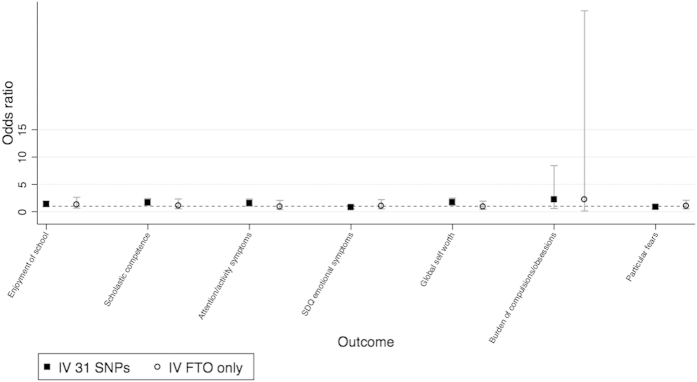
Testing invalidity of IV assumptions: associations of two instrumental variables using distinct SNP subsets, of binary outcomes. Odds ratio between outcome groups, for a 1 SD change of log BMI aged 8. Comparison between the SNP subsets: (1) 31 SNPs (excluding FTO SNP) and (2) the FTO SNP only. IV estimate of effect using two-stage least squared regression of log BMI at age 8 as the exposure. Graphical illustration of the results in Table 5. Categories for binary variables are given in Supplementary Table 7.

The 31 SNP score and *FTO* allele were both strong instruments for log BMI at age 8. A 1 SD increase of the 31-SNP score was associated with a 0.146 standard deviation increase in log BMI at age 8 (95% CI: 0.12, 0.17, F = 112.70). A 1 SD increase of *FTO* was associated with a 0.074 standard deviation increase in log BMI at age 8 (95% CI: 0.05, 0.10, F = 28.18). We found little evidence of pleiotropy, linkage disequilibrium or population stratification as the tests with the *FTO* and 31-SNP scores were highly consistent (Figs 4 and 5 and Table 5). We found evidence using the Hansen tests of differences between the estimated effects of BMI using each instrument for 5 outcomes: apolipoprotein AI, apolipoprotein B, insulin, leptin and the emotional symptoms score (Table 6). This may be due to chance, or alternatively may suggest that the genetic variants related to BMI have pleiotropic or heterogeneous effects on these outcomes.

Table 1 shows the associations of *FTO* and the 31-allele score respectively, with BMI across childhood. We found evidence of an inverse association of *FTO* with BMI in early childhood, as previously suggested^39^. For instance, an increase of 1 BMI increasing *FTO* allele was associated with a 0.059 decrease of log BMI at age 1 year 8 months (95% CI: −0.096, −0.022). In contrast, the 31-allele score was positively associated with BMI at all ages measured.

### Sensitivity analyses

**Figure 6:**
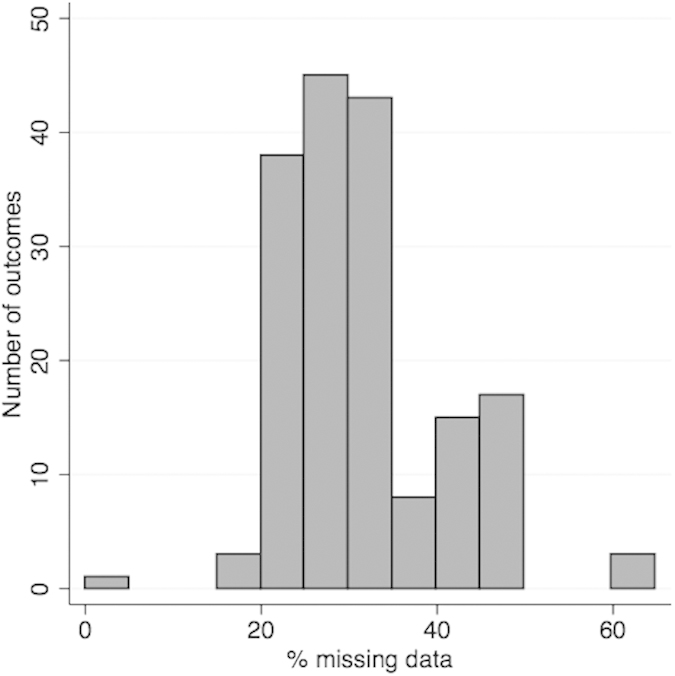
Distribution of the percentage of missing data, in our 8,121 sample, across the 172 outcomes.

As shown in Fig. 6, the outcomes had varying numbers of missing values, which means there were differences in statistical power across outcomes. However, the ranking of our main analysis is highly correlated with the ranking of the imputation dataset (Spearman’s rank correlation of 0.919 (P < 0.001)). Results using the imputed dataset are given in Supplementary Table 4.

We looked for further evidence of the associations identified during our main analyses by repeating the two-stage least squares IV analysis of the second stage using a 52-SNP BMI allele score instead of the 32-SNP score. A standard deviation increase in the 52-SNP BMI allele score was associated with a 0.080 standard deviation increase in log BMI at age 8 (95% confidence interval (CI): 0.05, 0.11, F = 33.21). The two-stage least squares IV analysis estimates using the 52-SNP BMI allele score were consistent with those using the 32-SNP score (see Supplementary Figures 1 and 2).

## Discussion

Epidemiologists have struggled to produce robust replicable evidence of the causal effect of risk factors^40^. Population geneticists have been extremely successful in using hypothesis-searching approaches to produce replicable associations. We have shown that it is possible to use a similar hypothesis-searching approach, using Mendelian randomization to highlight the strongest effects of an exposure in a large sample of individuals.

We used a Mendelian randomization analysis to screen for potentially causal effects. Our stage 1 analysis tested the association of the BMI allele score directly with each of the outcomes. Identifying known effects with this approach validates the use of this score as an instrument for life-long BMI. The BMI allele score was most strongly associated with leptin, which is produced in adipose tissue and is involved in satiation^41^. This result is consistent with previous research^28^. Also consistent with previous studies, we identified effects of BMI on the following metabolic traits: glucose, insulin, interleukin-6, systolic blood pressure^24^, low and high density lipoprotein cholesterol, triglycerides, and C-reactive protein^21^,^27^,^28^,^31^,^34^,^42^,^43^. The BMI allele score was also strongly inversely associated with age at menarche. Previous observational studies have reported that age at menarche is inversely associated with BMI^44^, and a recent study also used Mendelian randomization to argue that BMI affects age at menarche^25^. We found a novel positive effect of BMI on a global self-worth score. We did not replicate the novel associations, as our aim in this work is to carry out a proof-of-principle analysis demonstrating our hypothesis-searching method. We found more associations using BMI aged 8 than with the BMI allele score. This may be due to reverse causation (because some of the “outcomes” actually affect BMI) or due to confounding, and this highlights the benefit of a Mendelian randomization analysis^6^. While this could be due to the lower power of tests with the allele score compared to observational BMI, when the stage 1 tests were repeated using an allele score composed of all 84 variants (combining the 52 and 32 SNP score) the number of results with p < 0.05 was comparable to that found using the 32 SNP score, (19 versus 21 respectively, test of proportions p = 0.737).

The observational estimates were consistently closer to the null than the IV estimates. This may be due to the winner’s curse because in the stage 1 we rank all 172 estimates of the allele score on the outcomes, such that the highest ranked are more likely to be higher than their respective true values because of the random variation of these sample estimates about their true values. The stage 2 estimates (also ranked by the stage 1 estimates) may also be affected by this winners curse because the stage 1 (direct) and stage 2 models are highly related. The estimates using the observational BMI exposure may not affected to the same extent as they are ranked by the results using the BMI allele score, with which it is not perfectly correlated. Alternatively, the IV estimates may be more extreme than the observational estimates because the allelic score is a measure for life-long BMI, so the effect on outcomes may be larger due to the cumulative effects of BMI across the life course.

The validity of our results depends on whether the instrumental variable assumptions hold, such that the genetic variants only affect the outcomes through BMI (the exposure). We ensured our BMI allele score was a strong instrument for BMI, and was not associated with common confounding variables such as sex and potential socio-economic confounders such as household social class. In contrast, BMI at age 8 was associated with confounders consistent with the social patterning reported previously^45^,^46^,^47^. The allele score was composed of 32 BMI-associated genetic variants. While a larger number of SNPs increases the power to detect associations, this also means that the third instrumental variable assumption may be more likely to be invalid for at least one genetic variant included in the allele score.

A number of mechanisms could invalidate the third instrumental variable assumption: that the genetic variants only affect the outcomes through BMI at age 8. These include genetic induced confounding through horizontal pleiotropy (where a locus affects several outcomes directly^9^), population stratification and linkage disequilibrium, each of which could add a causal path from the IV to an outcome which was not mediated via BMI. The effect estimates when using two independent instruments (*FTO* and the remaining 31 variants) were consistent providing evidence against pleiotropy because it is unlikely that two independent instruments suffer the same pleiotropic effects. Furthermore, the IV estimates using the 52-SNP allele score were consistent with the IV estimates of our 32-SNP score. However, we found evidence of heterogeneity when testing the 32 SNPs individually using Hansen tests, for 5 of the 21 ‘top’ results. This may indicate either the core or point identifying IV assumptions are invalid. Any biases introduced by violations of these assumptions may be amplified due to the low power of the individual SNPs. This is because these weak instruments account for only a small proportion of the variance of BMI, such that their effect through BMI is small compared with the strength of the association through one of these alternative pathways^13^. Further tests to investigate heterogeneity could be performed in follow up analyses. For instance, plotting the effect of each SNP on BMI against the effect of each SNP on a particular outcome may identify SNPs causing the heterogeneity, where they deviate from this linear association that should pass through the plot origin^48^. Repeating the Hansen tests with these SNPs removed can demonstrate if they are driving the reported heterogeneity.

The stage 1 analysis estimated the association of the BMI allelic score and each outcome, rather than providing an estimate of the effect size, as is estimated in the stage 2 IV analysis. While an estimate of the effect size is generally preferable, the stage 1 tests are important to consider because they only depend on the core IV assumptions, whereas the stage 2 tests also require point identifying assumptions. Also, some of the ways the IV assumptions can become invalidated are circumvented in the stage 1 tests. This is because the stage 1 test requires the exposure to be defined, but a variable representing this exposure is not actually used in the test of association. This is useful because the BMI allelic score is a measure of lifelong risk of increased BMI, but we only have measures of observational BMI at a set of discrete time points, rather than a composite measure representing observational lifelong BMI. The use of BMI at a single time point, at age 8, is a valid exposure if all pathways through BMI at all other ages (prior to measurement of the outcome) also pass through BMI at age 8. If this is not the case (and this is likely) then the IV assumptions are false. For example, we found a different effect of the *FTO* SNP and 31 SNP allele score on BMI in early childhood, which indicates that these variants affect BMI through different pathways. Any pathway from the genetic variants to an outcome through BMI at an age other than age 8 would invalidate the instrumental variable assumptions. This is not a problem for our stage 1 tests, as we need only specify the exposure as ‘lifelong BMI up to the point of outcome measurement’. Furthermore, this removes the issue of measurement error in the observed exposure variable since it is not actually used in the model.

We now discuss some further limitations of our analysis. We tested only for linear relationships and hence it is possible that non-linear relationships exist. We used an inverse rank normal transformation, which may not be appropriate for numeric outcomes with only a small number of values, as the rank within each set with the same value is randomly generated, and this may add noise to the data. Ranking results means that we should expect the true strength of associations to be less than we reported, due to the winner’s curse. This means that the effect sizes are not reliable and need replicating in a hypothesis driven manner. However, conventional epidemiological studies also suffer this due to flexibility in study design, where several methods may be used in turn to examine a particular relationship and the strongest result reported^49^. The size of an effect estimate may be reduced due to developmental compensation (or canalisation) where a foetus may develop to protect itself from the adverse effects of a particular polymorphism that is expressed during foetal development. This protection may continue throughout the life course such that a high BMI will have fewer health implications, and our reported associations may be reduced^8^,^9^. Dynastic effects, where the outcome trait of the child is also affected by the parental exposure caused by the parental genotype, can also affect the size of an effect estimate. The effect may be exaggerated because the child has greater exposure, or reduced if the parent’s genotype creates an adverse environment during pregnancy from which the foetus then develops to protect against.

Our dataset included the most complete version of each repeated measure, which was usually at the earlier time point. While this may improve the statistical power of our tests, this benefit may be offset by the reduction of power because associations are often less pronounced at younger ages (as shown in Table 1 for BMI). Mendelian randomization analyses have low statistical power compared to conventional observational analyses, because genetic variants typically only explain a small proportion of an exposure’s variance. Although we used a combined allelic score to maximize the power from the genetic predictors, some associations may not have been detected due to a lack of power. Furthermore, performing multiple tests reduces the statistical power as we need to account for the number of independent tests we performed. The varying degrees of missingness of the outcome variables means: (1) it is possible the associations are biased if the outcome data are not missing at random (conditional on the variables in the model, i.e. observed BMI or the allelic score), and (2) the ranking may be affected by differences in power amongst the outcomes, including false negative results where the power is too low to detect an association. Using the time point with the largest available sample sizes for each trait reduces the risk of bias due to missingness.

Traditionally, hypothesis-driven studies, where many hypotheses are tested independently by several research teams, suffer the issues of multiple testing and selection bias from the researcher choosing which hypothesis to test and the methods to use, as well as publication bias^50^. A large proportion of null findings are unpublished such that it is not possible to determine the true probability the reported result would occur by chance. This is a most problematic form of multiple testing because we cannot know how many and what associations have be tested. In contrast, by searching for hypotheses in a single study we are able to report the results of all analyses, including ‘null’ results, so that our work does not contribute to this publication bias. We have provided the results of all stage 1 tests in the Supplementary Material. We presented unadjusted and Bonferroni corrected P values, and estimated a false discovery rate of 0.571, such that 6 of the 14 associations we found with a P value <0.05 (excluding our validation set) may indicate causal relationships between BMI and these outcomes. Given the high degree of confounding in observational data, the adjusted P values and false discovery rate are likely to be conservative estimates, because they both account for the number of independent tests, but the outcomes in our dataset are not independent. We also provide permutation testing P values that are an appropriate way to assess the results as this method implicitly accounts for the number of tests performed. A result is less likely to achieve a rank of 1 by chance alone as the number of tests increases. The P values of the Bonferroni and permutation testing were very different, highlighting the conservative nature of a Bonferroni correction when outcomes are not independent. Furthermore, we investigated an alternative Bonferroni correction (see Supplementary Material) that instead accounts for the number of independent variables, and this found 12 associations with P < 0.05 out of 128 independent outcomes (see Supplementary Material).

A Bonferroni correction should be used when concerned with the global hypothesis, such that the researcher wants to control the probability that at least one test is incorrectly shown to have an association by chance, known as a false positive finding^51^. Bonferroni corrections use a more stringent threshold such that while the number of false positive findings is lower, the number of ‘true’ associations that are not identified (because their associated P values are above the Bonferroni corrected threshold) is higher. This is not helpful for hypothesis searching studies because we may then miss potentially important associations. Also, the cost of a false positive association is lower in hypothesis searching studies compared with traditional epidemiological studies because the results will be followed up with a further analysis rather than claimed to be a definitive result. In a hypothesis-searching study the researcher may be happy to follow up *n* tests knowing that *m%* of these may be false positives, such that it may be more appropriate to control the false discovery rate. This false discovery rate can be adjusted by changing the P value threshold.

The observational associations reported by previous EWAS studies may be caused by bias or confounding and do not provide reliable evidence of causation. We have used Mendelian randomization to search for true, causal relationships. Our analyses with observational BMI across childhood found a much larger number of strong associations, a distinction that has been previously reported^6^. EWAS studies may be worthwhile to test observational relationships, which can then be followed up with a Mendelian randomization analysis. However, observational associations may be weaker than the true causal effect because masked confounding and measurement error can move associations towards the null^52^. The pheWAS approach has been previously used to identify associations between a set of genetic variants and a set of phenotypic variables^18^. Our approach extends the pheWAS approach in order to identify potentially causal associations. While pheWAS test the association of individual SNPs with observed phenotypes, we use an allelic score composed of variants known to be associated with a particular risk factor as an instrumental variable.

**Figure 7:**
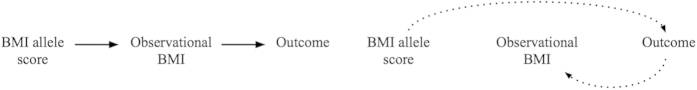
Graphs illustrating two possible causal pathways to explain associations of the BMI allele score with the outcomes. Abbreviations: BMI, body mass index. Left graph represents the intended pathway we have investigated, where the BMI allele score is an IV for BMI and the variants affect the outcome solely through observed BMI in childhood. The right graph represents the alternative causal pathway, where the allelic score affects BMI indirectly through the outcome variable. It is possible that these two pathways both occur for a given outcome, such that the graph would become cyclical.

While our approach provides evidence of causality, the direction of this causal effect is less clear, as illustrated in Fig. 7. Instead of our hypothesized relationship, it is possible that the allele score actually directly affects the “outcome”, which in turn affects BMI, our “exposure” variable. For instance, as leptin is involved in satiation it is possible that the BMI allelic score, or a subset of variants of which it is composed, affects BMI through leptin rather than vice versa. Currently, our understanding of the biological effects of these variants are often not sufficient to have certainty over the direction of the mechanism of action. Whilst it is not possible to directly test this, this can be investigated by comparing the effect estimates of independent instruments, as if two instruments affect the “outcome” through the “exposure” the estimated effect of BMI on an outcome should be consistent across different variants.

We believe hypothesis-searching with Mendelian randomization is a valuable first step towards identifying causal effects, without specifying a particular hypothesis a priori. As with GWAS, the results need to be followed up with further analyses on independent data. While replication is sufficient to determine if a GWAS association is robust, MR-pheWAS follow-up studies also need to determine if the association is due to a causal effect, such that causal inference can be made. Replication of an association between an allele score and a phenotype is not enough to infer causality because this association could be due to an effect of the “outcome” on the “exposure” as just discussed, or because the instrumental variable assumptions are invalid.

Where possible it would be informative to test the association of an allelic score of an “outcome” (e.g. leptin) with observational BMI, to further elucidate causality through a bi-directional analysis^9^. To ensure associations found are not due to reverse causation (where the “outcome” has a causal effect on the “exposure”) each allelic score should be composed of variants that have a strong association only with the “exposure” in this bi-directional analysis^53^. Also, identifying homogeneity of effects between SNPs can provide evidence for a direction of effect. For instance, suppose the IV estimates of the effect of BMI on an “outcome” using the BMI SNPs individually all imply a similar effect estimate, whereas the IV estimates of the effect of the “outcome” on BMI using each of “outcome” SNPs imply either heterogeneous or no effects, then this may suggest that BMI affects the “outcome”.

In this work we have demonstrated a hypothesis-searching approach to identify potentially causal effects of a risk factor, using BMI as an exemplar. Unlike traditional hypothesis driven approaches we test the association with a large, randomly selected set of phenotypes, rather than specifying a hypothesis to test a priori. We have found that observational BMI was associated with a large number of phenotypes, illustrating the problematic nature of observational tests due to the confounding prevalent between observational phenotypes. These associations in observational data do not indicate causality. In contrast, our genetic instrument was associated with fewer phenotypes because (subject to instrumental variable assumptions) its constituent alleles are not associated with confounding factors, and the causal direction can be investigated because the genome exists prior to the observed phenotypes. We used a set of positive controls to validate the use of this hypothesis-searching method.

This scalable and systematic approach can be repeated with Mendelian randomization variables of other exposures, in order to gradually determine the causal structure of an otherwise complex network. As discussed, associations identified with this type of analysis would need further investigation to validate the relationship through replication studies and elucidate the direction of causality.

## Methods

### Study population

The Avon Longitudinal Study of Parents and Children (ALSPAC) is a prospectively collected pregnancy cohort that recruited pregnant women with expected delivery dates between April 1991 and December 1992 from Bristol, UK (see^54^,^55^,^56^ for the study details). The study was carried out in accordance with the ethical standards of the Helsinki Declaration, and ethical approval for data collection was obtained from the ALSPAC Law and Ethics Committee and local research ethics committees. Informed consent was obtained from all subjects.

A total of 9,912 ALSPAC children were genotyped using the Illumina HumanHap550 quad genome-wide SNP genotyping platform by the Wellcome Trust Sanger Institute, Cambridge, UK and the Laboratory Corporation of America, Burlington, NC, USA. Individuals were excluded from further analysis on the basis of having incorrect sex assignments; minimal or excessive heterozygosity (<0.320 and >0.345 for the Sanger data and <0.310 and >0.330 for the LabCorp data); disproportionate levels of individual missingness (>3%); evidence of cryptic relatedness (>10% IBD) and being of non-European ancestry (as detected by a multidimensional scaling analysis seeded with HapMap 2 individuals, EIGENSTRAT analysis revealed no additional obvious population stratification and genome-wide analyses with other phenotypes indicate a low lambda). The resulting data set consisted of 8,365 individuals and 488,311 autosomal SNPs. SNPs with a minor allele frequency of <1% and call rate of <95% were removed. Furthermore, only SNPs which passed an exact test of Hardy–Weinberg equilibrium (p > 5 × 10^−7^) were considered for analysis. Of these 8365 individuals, 5819 had BMI data, and 4251 had CRP and LDLc levels measured. Known autosomal variants were imputed with MACH 1.0.16 Markov Chain Haplotyping software, using CEPH individuals from phase 2 of the HapMap project (HG18) as a reference set (release 22).

After quality control assessment and imputation the data set consisted of 8,365 non-related children of European descent with 2,608,006 SNPs available for analysis. Of these 8,365 we removed 244 individuals with no data for all outcomes giving a sample size of 8,121. We restricted our analysis to individuals of white European ethnic origin to reduce the potential for population stratification, which could confound associations between the BMI allele score and the outcomes.

### BMI allele score

We created an allele score of the BMI variants, constructed using a weighted sum of 32 loci known to be associated with BMI (listed in Supplementary Table 1). The weights were generated from the effect size of BMI associated SNPs found in a large GWAS^57^. This GWAS did not include the ALSPAC study^58^. We constructed the score in terms of the number of BMI-increasing alleles so that a higher score corresponds to a higher BMI (see Supplementary Material for calculation).

### Outcomes

We compiled a set of 172 continuous variables from the ALSPAC dataset, comprising a range of variables recorded between birth and 15 years old, including primary measures (from questionnaires or focus clinics) and also derived variables. The dataset was compiled by selecting a set of complete clinic assessment based data files from the ALSPAC cohort, each corresponding to a separate measurement event. The intention is to include a random subset of available clinic measures, rather than select variables where we have an a priori interest or evidence in their association with BMI. These were processed in turn to reduce the size of the dataset by manually removing variables where multiple similar variables were found. We did this by including a composite score measure where available (and removing its component phenotypes) or keeping only one measure from each similar group of variables. This delivered a diverse range of 160 randomly selected variables at a range of time points to give a rich outcome dataset (given in Supplementary Table 3). We also included a selected set of outcomes, as we need to ensure that the dataset contains variables both with and without previous evidence of an association with BMI, such that we can validate our screening approach. We therefore included 12 outcomes, previously suggested to be associated (perhaps causally) with BMI: glucose^31^, insulin^31^, leptin^28^, age at menarche^25^, systolic blood pressure^24^ and C-reactive protein^28^,^34^, intelligence and attainment measures (Wechsler Intelligence Scale for Children (WISC), Diagnostic Analysis of Nonverbal Accuracy (DANVA), and literacy scores (2 phenotypes))^59^, lung function^60^ and the Home Observation for Measurement of the Environment (HOME) score^61^. Further details are given in Supplementary Table 2. Where outcomes were available at multiple time points we used the most complete measure. We removed values coded as missing, and refer to this as the original dataset.

### Statistical methods

We performed all analyses using Stata v11.2 (StataCorp LP, 2009; College station, TX, USA)^62^. Our main analysis, to search for associations, was a two-stage process. The first stage involved a large-scale analysis to screen for associations of the BMI allele score with all outcomes in our dataset. The second stage followed up ‘top’ associations identified in the first stage, with an IV analysis and sensitivity analysis assessing the degree of pleiotropy.

In the first stage we began by transforming the outcome variables in order to harmonise this dataset, such that a single analytical approach can be applied in the subsequent BMI score screening step. We used a rank-based inverse normal transformation to ensure all outcomes were normally distributed. We tested the associations of the BMI score with all transformed outcomes, using univariate linear regression analysis, with robust standard errors (the robust option). We ordered the resulting associations by P value to rank the associations from strongest to weakest (where a rank of 1 denotes the strongest result). The rank position gives an indication of the relative strength of associations of the allelic score with the outcome variables. We identified associations of outcomes and allele score with, as an illustration, a nominal P < 0.05 and took these forward for further tests in the second stage analysis.

In addition to the P values of these tests we report Bonferroni adjusted P values calculated by multiplying the P values by 160 to account for the number of tests performed. We exclude the validation set from these calculations as we have selected these phenotypes based on prior knowledge. We determine the proportion of our top results that are expected to be false positives, the false discovery rate (calculated as the expected number of results with P value < 0.05 by chance alone (160 × 0.05) divided by the number of results found with a P value < 0.05). We also report alternative permutation P values - the probability that an outcome at rank *i,* would be found at rank *j* where *j* ≤ *i* given there is no association between the BMI score and all outcomes. These are estimated using permutation testing, and we again exclude the validation set from this analysis. We permute the values of each outcome variable across participants and repeat the stage 1 analysis, performing linear regression of the BMI score on each outcome and generating a ranking of associations. We repeat this 5,000 times to derive an empirical distribution of the rank position of each outcome. For each outcome at rank *i* we report the proportion of these tests where the outcome is found at rank *j* where *j* ≤ *i*. This gives a P value that accounts for tests with all 160 randomly selected outcomes in the dataset. We also perform a sensitivity analysis to demonstrate the conservative nature of the standard Bonferroni correction (see Supplementary Material).

In the second stage of our analysis we tested each stage 2 outcome (that had an association with p < 0.05 in stage 1) with a formal instrumental variable analysis using two-stage least squares regression (the Stata ivregress command). Although the BMI allele score is a risk factor for lifelong BMI we did not observe lifelong BMI and so instead use this score to estimate the effect of BMI at a single time point, at age 8, on the outcomes. We log-transformed BMI at age 8 so that its distribution was approximately normal. We used the original outcome dataset (rather than the inverse normal transformed version) and transformed any variables with skewed distributions (identified visually) to give distributions that were approximately standard normal (with mean of zero and standard deviation of one). We converted outcomes with distributions that were not normal and not right skewed to binary variables with approximately equal numbers in each group. We used linear and logistic regression for the second stage of the instrumental variable analysis for normally distributed and binary outcomes respectively. Finally, we also tested the associations of observational (log-transformed) BMI at age 8 using the same protocol with the 172 outcome variables, for comparison.

### Testing validity of the instrumental variable assumptions

As discussed in the introduction, Mendelian randomization tests require the IV assumptions to be satisfied. These assumptions are; (1) the genetic IV is associated with the exposure (observational log BMI at age 8), (2) the genetic IV only affects the outcome through its effect on the exposure, and (3) the genetic IV is independent of all factors confounding the association between BMI and the outcomes. We used univariate linear regression (the Stata regress command) to test the strength of the BMI allele score as an instrument for observational BMI, where a larger F-statistic implies greater power^63^. We cannot directly test assumptions 2 and 3, but we can look for evidence that the assumptions do not hold. If the 3 core instrumental variable assumptions hold, and either of the point identifying assumptions hold, the estimated effect of BMI on an outcome should be consistent across different variants. We explored this by comparing the results using two independent instrumental variables: (1) *FTO* (the SNP most strongly associated with BMI), and (2) the remaining 31 variants (we refer to this as the 31-allele score). FTO explains around a quarter of the variance of BMI explained by the other 31 combined (31 variants were associated with BMI r^2^ = 0.0215, FTO was associated with BMI r^2^ = 0.0055). Furthermore, we tested the strength of the associations of each instrument to ensure they were both strongly associated with BMI.

We performed further tests with the stage 2 outcomes (those below P < 0.05 in stage 1) to look for evidence that the variants were not valid instruments. When there are more instruments (genetic variants) than dependent variables (risk factors), an instrumental variable analysis is over-identified, and we can test for differences between estimates based on each of the variants, using over-identification tests. We estimated the effects of BMI using the 32 individual SNPs with the continuously updating estimator (CUE) via generalized method of moments (GMM), and tested for heterogeneity in these effects using Hansen tests^64^. The null hypothesis states that there is no evidence of differences in the IV effect estimates between different variants. Thus rejection of this test suggests there are differences between the estimates based on each of the variants. This may suggest that the instrumental variable assumptions do not hold, for example if the effects of the variants are not solely mediated through BMI.

### Sensitivity analyses

We present results using the original data as our main analyses, where the sample size for each test varies depending on the outcome. This creates a potential for differences in P values to be caused by differences in sample size, or bias due to missing data. In order to assess the impact of this we repeated our analysis using an imputed dataset. We used multiple imputation with chained equations to create 20 imputed datasets of all 8,121 individuals and 172 variables in the original dataset (details of the imputation methods^65^ can be found in the Supplementary Material). We compared the ordering of the outcome variables by P value across the original and imputed versions using Spearman’s rank correlation.

We performed a further sensitivity analysis to test for further evidence of the associations identified in our IV analysis. We used 52 novel loci associated with BMI, identified in the largest GWAS to date^66^, to construct a weighted allele score, and refer to this as the 52-SNP allele score (SNPs included are given in Supplementary Table 8). These loci are independent to those included in our analyses. We repeat the IV analysis to determine if the associations using this independent IV are consistent with our original results.

## Additional Information

**How to cite this article**: Millard, L. A. C. *et al.* MR-PheWAS: hypothesis prioritization among potential causal effects of body mass index on many outcomes, using Mendelian randomization. *Sci. Rep.* **5**, 16645; doi: 10.1038/srep16645 (2015).

## Supplementary Material

Supplementary Materials

## Figures and Tables

**Table 1:** Associations of BMI allele score with BMI across childhood.

Number with measured BMI	Mean age at BMI measurement	SD increase of log BMI during childhood per 1 SD increase of BMI allele score with 32 SNP variants			SD increase of log BMI during childhood per 1 SD increase of FTO allele dosages			SD increase of log BMI during childhood per 1 SD increase of BMI allele score with 31 SNP variants (FTO removed)		
		SD change	95% CI	F-statistic^a^	SD change	95% CI	F-statistic^a^	SD change	95% CI	F-statistic^a^
6,601	7.48 wks	0.016	−0.01, 0.04	1.65	−0.018	−0.04, 0.01	2.12	0.025	0.00, 0.05	4.22
6,282	40.50 wks	0.032	0.01, 0.06	6.36	−0.029	−0.05, 0.00	5.19	0.047	0.02, 0.07	14.10
5,797	1.69 yrs	0.036	0.01, 0.06	7.42	−0.041	−0.07, −0.02	9.66	0.056	0.03, 0.08	18.78
5,430	3.71 yrs	0.068	0.04, 0.09	25.29	−0.004	−0.03, 0.02	0.10	0.075	0.05, 0.10	31.39
6,076	7.57 yrs	0.142	0.12, 0.17	125.00	0.049	0.02, 0.07	14.88	0.133	0.11, 0.16	109.75
5,087	8.68 yrs	0.163	0.14, 0.19	140.66	0.074	0.05, 0.10	28.18	0.146	0.12, 0.17	112.70
5,623	10.68 yrs	0.175	0.15, 0.20	175.27	0.090	0.06, 0.12	45.96	0.150	0.12, 0.18	128.80
5,116	12.80 yrs	0.170	0.14, 0.20	151.21	0.082	0.05, 0.11	35.28	0.150	0.12, 0.18	116.58
4,746	13.83 yrs	0.167	0.14, 0.19	134.07	0.074	0.05, 0.10	26.78	0.150	0.12, 0.18	107.05
4,174	15.45 yrs	0.158	0.13, 0.19	106.35	0.071	0.04, 0.10	21.60	0.142	0.11, 0.17	84.49
2,665	17.04 yrs	0.176	0.14, 0.21	83.94	0.086	0.05, 0.12	20.14	0.153	0.12, 0.19	63.13

Abbreviations: BMI, body mass index; CI, confidence interval; SD, standard deviation; wks, weeks; yrs, years. All BMI variables are log transformed. BMI calculated as weight (kilograms) divided by height (metres squared). Based on the original data of 8,121 participants (variable sample size per BMI measurement).

^a^F-statistic: Measure of strength of association between exposure and outcome.

**Table 2:** Association of BMI allele score and observational BMI at age 8 with potential confounders of BMI.

Potential confounder	Sample size	SD change of BMI allele score for a 1 SD change of confounder			Sample size	SD change of log BMI age 8 for a 1 SD change of confounder		
		SD change	95% CI	P value^d^		SD change	95% CI	P value^d^
Maternal education^a^								
Less than O-level	7,306		Reference	0.871	4,735		Reference	0.001
O-level		−0.02	−0.08, 0.04			−0.05	−0.13, 0.03	
A-level		0.03	−0.04, 0.09			−0.05	−0.13, 0.03	
Degree or above		−0.03	−0.11, 0.04			−0.16	−0.25, −0.07	
Sex								
Female	8,121		Reference		5,087		Reference	
Male		0.00	−0.05, 0.04	0.828		0.13	0.07, 0.18	<0.001
Household social class^b^								
Class I (professional)	6,929		Reference	0.776	4,554		Reference	0.213
Class II		0.01	−0.06, 0.08			0.10	0.02, 0.18	
Class III (non-manual)		−0.01	−0.08, 0.07			0.09	0.00, 0.19	
Class III (manual)		−0.01	−0.11, 0.08			0.10	−0.02, 0.21	
Class IV/V (manual)		0.02	−0.11, 0.14			0.07	−0.09, 0.23	
Parity^c^								
0	7,408		Reference	0.850	4,741		Reference	0.789
1		−0.03	−0.08, 0.02			−0.03	−0.10, 0.03	
2		0.01	−0.05, 0.07			0.01	−0.07, 0.08	
Mother smoking in pregnancy								
No	7,484		Reference		4,788		Reference	
Yes		0.02	−0.03, 0.08	0.444		0.18	0.11, 0.25	<0.001

Abbreviations: SD, standard deviation; CI, confidence interval; BMI, body mass index.

^a^Maternal education: O-level (ordinary level) exams are taken in different subjects usually at age 15–16 at the completion of legally required school attendance, equivalent to today’s General Certificate of Secondary Education); A-level (Advanced-level) exams are taken in different subjects usually at age 18).

^b^Household social class: The mother recorded the occupation of both herself and her partner in a questionnaire at 32 weeks gestation, which were used to allocate them to social class groups using the 1991 Office of Population, Censuses and Surveys classification; the highest class of the mother and her partner was used in analysis.

^c^Parity: Obtained from obstetric records. Mothers with parity of two or more were grouped into a single category.

^d^P values for linear trend given if more than two ordinal categories.

**Table 3:** Ranking by association strength (P value) of the stage 1 tests with p < 0.05: Outcome associations with BMI allele score for original dataset.

Rank	Outcome variable (original data with variable N)	SD change of inverse normal transformed outcome for a 1 SD change of BMI allele score				
		Sample size	SD change	95% CI	P value (adjusted P value)^a^	Permutation P value^b^
1	Leptin, 9^c^	4,249	0.138	0.11, 0.17	<0.001	–
2	CRP, 9^c^	4,250	0.083	0.05, 0.11	<0.001	–
3	Age menarche^c^	2,946	−0.083	−0.12, −0.05	<0.001	–
4	HDL, 9	4,250	−0.067	−0.10, −0.04	<0.001 (0.002)	0.008
5	SBP, 7^c^	6,013	0.049	0.02, 0.07	<0.001	–
6	IL6, 9	4,240	0.053	0.02, 0.08	0.001, (0.091)	0.010
7	Enjoyment of School Score, 4	5,807	0.041	0.02, 0.07	0.002, (0.255)	0.022
8	Self Esteem: Scholastic Competence, 8	5,222	0.042	0.02, 0.07	0.002, (0.323)	0.025
9	Apolipoprotein B, 9	4,250	0.043	0.01, 0.07	0.005, (0.788)	0.028
10	Triglycerides, 9	4,250	0.042	0.01, 0.07	0.006, (0.970)	0.037
11	VLDL, 9	4,250	0.042	0.01, 0.07	0.006, (1)	0.048
12	Apolipoprotein al, 9	4,250	−0.038	−0.07, −0.01	0.012, (1)	0.046
13	Insulin, 15^c^	2,859	0.047	0.01, 0.08	0.012	–
14	Attention/activity symptoms score, 11	4,541	0.037	0.01, 0.07	0.013, (1)	0.055
15	SDQ emotional symptoms score, 6	5,748	−0.030	−0.06, 0.00	0.022, (1)	0.063
16	Hygiene Score, 4	6,231	0.028	0.00, 0.05	0.024, (1)	0.070
17	Self Esteem: Global Self Worth Score, 8	5,214	0.031	0.00, 0.06	0.025, (1)	0.079
18	FVC: lung function, 8^c^	5,276	0.030	0.00, 0.06	0.030	−
19	Burden of compulsions/obsessions score, 7	5,684	0.028	0.00, 0.05	0.031, (1)	0.080
20	Particular fears score, 7	5,734	−0.028	−0.05, 0.00	0.033, (1)	0.086
21	Glucose, 15^c^	2,862	0.041	0.11, 0.17	0.038	–

Abbreviations: BMI, body mass index; CI, confidence interval; SD, standard deviation; VLDL, very low density lipoprotein; IL6, interleukin 6; SBP, systolic blood pressure; HDL, high density lipoprotein; VLDL, very low density lipoprotein; SDQ, Strengths and Difficulties Questionnaires; LF, lung function; FVC, forced vital capacity. Full names of variables are given in Supplementary Table 3. All outcomes are transformed to normal distributions using a rank-based inverse normal transformation. Exposure and outcome variables are standardised. Outcome as dependent variable, BMI allele score as independent variable.

^a^Adjusted P values are adjusted for the 160 tests performed using the Bonferroni correction: p_corrected_ = p_original_·160. Adjusted P values greater than 1 are rounded to 1. Outcomes in validation set are excluded from Bonferroni correction.

^b^Permutation P values are generated with permutation testing. Null hypothesis: The outcome variable at rank i in this table would be ranked lower than *i* if no association was found with the allelic score. We exclude outcomes in validation set such that such that, for instance *i* = *1* for HDL because all three outcomes ranked higher than HDL are in the validation set).

^c^Variables that are in the ‘validation set’, that were chosen for inclusion using apriori knowledge of their association with BMI. Adjusted P value are not given for these are they are not part of the main outcome dataset.

**Table 4:** Ranked outcome associations with BMI allele score for original dataset, for results with P < 0.05.

Rank	Outcome variable (original data with variable N)	Sample size	IV estimate of SD change of outcome for a 1 SD change of log BMI age 8^a^			SD change of outcome for a 1 SD change of log BMI (observational, age 8)			P value^e^
			Test statistic	95% CI	P value (adjusted P value^b^)	Test statistic	95% CI	P value (adjusted P value^b^)	
Linear regression of continuous, normally distributed outcomes. Test statistic is the mean difference (SD) per 1 SD greater log BMI age 8 or percentage difference per 1 SD greater log BMI age 8 for log transformed outcomes^c^									
1	Leptin, 9^f^	3,381	0.922	0.77, 1.07	<0.001	0.688	0.66, 0.71	<0.001	0.002
2	CRP, 9^f^	3,382	0.432	0.25, 0.62	<0.001	0.367	0.33, 0.40	<0.001	0.484
3	Age menarche^f^	2,186	−0.485	−0.70, −0.27	<0.001	−0.298	−0.34, −0.26	<0.001	0.080
4	HDL, 9	3,382	−0.466	−0.66, −0.27	<0.001 (<0.001)	−0.303	−0.34, −0.27	<0.001 (<0.001)	0.096
5	SBP, 7^f^	4,641	0.305	0.13, 0.48	0.001	0.312	0.28, 0.34	<0.001	0.940
6	IL6, 9	3,372	0.234	0.04, 0.43	0.018 (2.852)	0.197	0.17, 0.23	<0.001 (<0.001)	0.709
9	Apolipoprotein B, 9	3,382	0.363	0.16, 0.57	<0.001 (0.080)	0.163	0.13, 0.20	<0.001 (<0.001)	0.048
10	Triglycerides, 9	3,382	0.310	0.11, 0.51	0.002 (0.341)	0.226	0.19, 0.26	<0.001 (<0.001)	0.392
11	VLDL age 9	3,382	0.310	0.11, 0.51	0.002 (0.343)	0.226	0.19, 0.26	<0.001 (<0.001)	0.393
12	Apolipoprotein al, 9	3,382	−0.243	−0.44, −0.05	0.016 (1)	−0.196	−0.23, −0.16	<0.001 (<0.001)	0.636
13	Insulin, 15^f^	2,285	0.299	0.06, 0.53	0.013	0.202	0.16, 0.24	<0.001	0.434
16	Hygiene Score, 4	4,335	0.209	0.01, 0.41	0.042 (1)	0.052	0.02, 0.08	0.001 (0.086)	0.118
18	FVC: lung function, 8^f^	4,869	0.162	0.00, 0.32	0.052	0.147	0.12, 0.18	<0.001	0.855
21	Glucose, 15^f^	2,288	0.334	0.02, 0.65	0.039	0.006	−0.04, 0.05	0.819	0.012
Logistic regression of binary outcomes. Test statistic is the odds ratio between outcome groups^d^ for a 1 SD increase in log BMI age 8									
7	Enjoyment of School Score, 4	5,808	1.344	0.98, 1.84	0.066 (1)	1.075	1.01, 1.14	0.025 (1)	0.700
8	Self Esteem: Scholastic Competence, 8	5,223	1.548	1.11, 2.16	0.010 (1)	1.004	0.95, 1.06	0.896 (1)	0.036
14	Attention/activity symptoms score, 11	4,542	1.399	0.96, 2.03	0.079 (1)	1.022	0.95, 1.10	0.570 (1)	0.166
15	SDQ emotional symptoms score, 6	5,749	0.856	0.62, 1.19	0.357 (1)	0.947	0.88, 1.01	0.095 (1)	0.543
17	Self Esteem: Global Self Worth Score, 8	5,215	1.536	1.10, 2.14	0.011 (1)	1.002	0.95, 1.06	0.932 (1)	0.046
19	Burden of compulsions/obsessions score, 7	5,685	2.258	0.74, 6.92	0.154 (1)	0.970	0.67, 1.27	0.842 (1)	0.507
20	Particular fears score, 7	5,735	0.893	0.65, 1.23	0.487 (1)	0.992	0.93, 1.05	0.792 (1)	0.519

Abbreviations: BMI, body mass index; CI, confidence interval; SD, standard deviation; IV, instrumental variable; VLDL, very low density lipoprotein; IL6, interleukin 6; SBP, systolic blood pressure; HDL, high density lipoprotein; SDQ, Strengths and Difficulties Questionnaires; FEF, forced expiratory flow; LF, lung function; FVC, forced vital capacity. Full names of variables are given in Supplementary Table 3. Figures 2 and 3 shows these results graphically. The ranks are based on tests of association of the BMI allele score with the outcome directly, for the 22 associations with a p < 0.05. We then perform IV analysis of these results, and it is the IV estimate that is given in this table. Exposure and outcome variables are standardized. Outcome as dependent variable, BMI allele score as independent variable.

^a^Continuous outcomes: Using Stata ivreg2 command (robust option) and BMI allele score as instrumental variable for log BMI age 8. First stage predicting log BMI at age 8 with the BMI allele score, and the second stage performs an unadjusted association of these log BMI age 8 predictions with the outcome. Binary outcomes: Using regress command (robust option) for first stage, and logistic command (vce(robust) option) for the second stage, to associate outcome with the predicted values of log BMI age 8 from the first stage.

^b^Adjusted P values: Adjusted for the 160 tests performed using the Bonferroni correction: p_corrected_ = p_original_·160. Adjusted P values greater than 1 are rounded to 1. Outcomes in validation set are excluded from Bonferroni correction.

^c^Log transformed outcomes, such that distributions approximately normal.

^d^Categories for binary variables given in Supplementary Table 7.

^e^P value of Durbin-Wu-Hausman test, comparing the effect estimates using the allelic score with the effect estimates using observational log BMI age 8. This uses a test for endogeneity (endog argument of ivreg2 Stata command).

^f^Variables that are in the ‘validation set’, that were chosen for inclusion using a priori knowledge of their association with BMI.

**Table 5:** Testing for invalidity of IV assumptions: associations of two instrumental variables for log BMI at age 8; using 31 SNPs (excluding FTO SNP) and only the FTO SNP respectively.

Outcome variable (original data with variable N)	IV estimate using 31 SNPs excluding FTO SNP^a^			IV estimate using FTO SNP^a^ only			Hansen P value^e^
	Test statistic	95% CI	P value^c^	Test statistic	95% CI	P value^c^	
Linear regression of continuous normally distributed outcomes. Test statistic is the mean difference (SD) per 1 SD greater log BMI age 8 or percentage difference per 1 SD greater log BMI age 8^d^							
Leptin, 9b,d	0.851	0.68, 1.02	<0.001	1.193	0.85, 1.54	<0.001	0.051
CRP, 9b,d	0.447	0.24, 0.66	<0.001	0.375	0.01, 0.74	0.042	0.735
Age menarche^b^	−0.443	−0.70, −0.19	0.001	−0.631	−1.05, −0.21	0.003	0.449
HDL, 9^d^	−0.517	−0.75, −0.29	<0.001	−0.271	−0.64, 0.10	0.153	0.275
SBP, 7^b^	0.290	0.10, 0.48	0.002	0.385	−0.05, 0.83	0.086	0.694
IL6, 9^d^	0.300	0.08, 0.52	0.008	−0.027	−0.41, 0.36	0.891	0.136
Apolipoprotein B, 9	0.281	0.05, 0.51	0.018	0.678	0.24, 1.12	0.003	0.098
Triglycerides, 9^d^	0.262	0.04, 0.49	0.023	0.496	0.09, 0.90	0.016	0.307
VLDL age 9^d^	0.261	0.04, 0.49	0.023	0.497	0.09, 0.90	0.016	0.303
Apolipoprotein al, 9	−0.275	−0.51, −0.04	0.021	−0.122	−0.50, 0.26	0.533	0.509
Insulin, 15b,d	0.214	−0.05, 0.48	0.116	0.634	0.08, 1.19	0.026	0.163
Hygiene Score, 4	0.241	0.02, 0.46	0.032	0.045	−0.44, 0.53	0.858	0.477
FVC: lung function, 8^b^	0.226	0.04, 0.41	0.016	−0.116	−0.49, 0.25	0.540	0.096
Glucose, 15^b^	0.423	0.06, 0.78	0.021	−0.018	−0.56, 0.52	0.949	0.161
Logistic regression of binary outcomes. Test statistic is the odds ratio between groups for a 1 SD increase in log BMI age 8							
Enjoyment of School Score, 4	1.349	0.95, 1.92	0.096	1.319	0.66, 2.64	0.435	0.897
Self Esteem: Scholastic Competence, 8	1.665	1.15, 2.41	0.007	1.122	0.54, 2.34	0.759	0.517
Attention/activity symptoms score, 11	1.534	1.01, 2.32	0.043	0.916	0.40, 2.07	0.832	0.882
SDQ emotional symptoms score, 6	0.816	0.56, 1.18	0.277	1.072	0.52, 2.22	0.852	0.815
Self Esteem: Global Self Worth Score, 8	1.723	1.19, 2.50	0.004	0.926	0.45, 1.93	0.837	0.357
Burden of compulsions/obsessions score, 7	2.241	0.60, 8.44	0.233	2.255	0.14, 36.76	0.568	0.734
Particular fears score, 7	0.864	0.61, 1.23	0.418	1.041	0.52, 2.10	0.910	0.901

Abbreviations: BMI, body mass index; CI, confidence interval; SD, standard deviation; IV, instrumental variable; VLDL, very low density lipoprotein; IL6, interleukin 6; SBP, systolic blood pressure; HDL, high density lipoprotein; SDQ, Strengths and Difficulties Questionnaires; LF, lung function; FVC, forced vital capacity. IV estimate calculated with ivregress and robust option (for robust standard errors). Categories for binary variables are given in Supplementary Table 7. Full names of variables are given in Supplementary Table 3. Figures 4 and 5 show these results graphically.

^a^FTO SNP is rs1558902.

^b^Variables that are in the ‘validation set’, that were chosen for inclusion using a priori knowledge of their association with BMI.

^c^P values are not adjusted for the multiple tests performed.

^d^Log transformed outcomes, such that distributions approximately normal.

^e^Hansen P value comparing effect estimates using 31 SNP score and FTO.

**Table 6:** Overidentification tests of IV using CUE.

Outcome variable (original data with variable N)	CUE^a^			
	Test statistic	95% CI	P value^b^	Hansen P value^b^
Linear regression of continuous normally distributed outcomes. Test statistic is the mean difference (SD) per 1 SD greater log BMI age 8 or percentage difference per 1 SD greater log BMI age 8^4^				
Leptin, 9^c^	0.934	0.74, 1.13	< 0.001	0.019
CRP, 9^c^	0.410	0.22, 0.60	< 0.001	0.606
Age menarche	−0.508	−0.82, −0.20	0.001	0.077
HDL, 9^c^	−0.447	−0.65, −0.24	< 0.001	0.388
SBP, 7	0.314	0.12, 0.50	0.001	0.456
IL6, 9^c^	0.250	0.07, 0.43	0.006	0.885
Apolipoprotein B, 9	0.339	0.04, 0.64	0.028	0.010
Triglycerides, 9^c^	0.245	0.04, 0.45	0.020	0.455
VLDL age 9^c^	0.245	0.04, 0.45	0.020	0.454
Apolipoprotein al, 9	−0.302	−0.53, −0.07	0.010	0.005
Insulin, 15^3^	0.781	0.28, 1.28	0.002	0.013
Hygiene Score, 4	0.299	0.08, 0.52	0.007	0.265
FVC: lung function, 8	0.111	−0.05, 0.27	0.180	0.670
Glucose, 15	0.312	−0.01, 0.63	0.059	0.877
Linear regression of binary outcomes. Test statistic is the change in probability that outcome has value 0 for a 1 SD increase in log BMI age 8				
Enjoyment of School Score, 4	0.008	−0.24, 0.25	0.949	0.309
Self Esteem: Scholastic Competence, 8	0.075	−0.10, 0.25	0.408	0.134
Attention/activity symptoms score, 11	0.174	−0.08, 0.43	0.184	0.702
SDQ emotional symptoms score, 6	0.066	−0.20, 0.33	0.621	0.046
Self Esteem: Global Self Worth Score, 8	0.087	−0.11, 0.28	0.374	0.097
Burden of compulsions/obsessions score, 7	0.035	−0.06, 0.13	0.475	0.587
Particular fears score, 7	−0.071	−0.28, 0.14	0.500	0.283

Abbreviations: BMI, body mass index; CI, confidence interval; SD, standard deviation; IV, instrumental variable; VLDL, very low density lipoprotein; IL6, interleukin 6; SBP, systolic blood pressure; HDL, high density lipoprotein; SDQ, Strengths and Difficulties Questionnaires; LF, lung function; FVC, forced vital capacity. Categories for binary variables given in Supplementary Table 7. Full names of variables are given in Supplementary Table 3. Tests use all 32 SNPs separately in the model.

^a^CUE: continuously updating estimator, with robust standard errors (robust option).

^b^P values are not adjusted for the multiple tests performed.

^c^Log transformed outcomes, such that distributions approximately normal.
