# Supplementary material for: MR-PheWAS: hypothesis prioritization among potential causal effects of body mass index on many outcomes, using Mendelian randomization
Source: Sci Rep. 2015 Nov 16;5:16645. doi: 10.1038/srep16645 (PMC4644974; doi:10.1038/srep16645)
Supplement: Supplementary Materials [file srep16645-s1.pdf]

MR-PheWAS: hypothesis prioritization among potential causal effects of body mass index on many outcomes, using Mendelian randomization

Louise A C Millard[* 1 2], Neil M Davies[1], Nic J Timpson[1], Kate Tilling[1], Peter A Flach[1 2], George Davey Smith[1]

* Corresponding author: louise.millard@bristol.ac.uk

Affiliations:

1. MRC Integrative Epidemiology Unit (IEU) at the University of Bristol, University of Bristol, Bristol
2. Intelligent Systems Laboratory, Department of Computer Science, University of Bristol, UK

Supplementary material

Calculating the BMI allele score

The BMI allele score was created using a weighted sum of allelic dosages, such that a higher score corresponds to a higher BMI:

$$\text{score}(i) = \sum_{l \in \text{loci}} \begin{cases} d_{l,i} \times \text{effect}_l : d_{l,i} \text{ is BMI increasing} \\ (2 - d_{l,i}) \times \text{effect}_l : \text{otherwise} \end{cases}$$

where d is the allelic dosage of individual i such that $0 \leq d \leq 2$, and effect_l is the effect size of loci l, scaled relative to the effect of FTO which has the largest effect size of these loci.

Imputation methods

The imputed dataset consisted of all 8,101 individuals and 172 variables in the original dataset. We used multiple imputation using chained equations (`ice` command in Stata), to impute missing values for all variables, and generated 20 imputation data sets (1). We used predictive mean matching (`match` option) for non-normal (or log-normal) variables because it does not assume normality, to prevent extrapolation beyond feasible values.

To inform the imputation we included additional socio-economic position (SEP) variables which may help to explain missingness: household social class, maternal education, smoking during pregnancy, and ethnicity. The purpose of this is to satisfy the missing at random (MAR) assumption of the imputation method; the probability of missingness does not depend on the missing data conditional on the observed data. We included the BMI allele score and all outcomes in our imputation, to inform the prediction of each outcome. The large number of variables in our dataset should also help to satisfy the MAR assumption, and the variable set should include variables predictive of both the variables and missingness of the variables (2).

Adjusting P values to account for the number of independent tests performed

We have presented both unadjusted and Bonferroni corrected P values. Given the high degree of confounding in observational data, the adjusted P values are likely to be a conservative estimate, as the Bonferroni correction accounts for the number of independent tests. A more appropriate adjustment would need to take into account the degree of dependence among the outcome variables, or use a set of outcomes that are independent. This 'independence adjusted' P value would sit between the unadjusted and Bonferroni corrected values. We performed a sensitivity analysis to investigate this, by creating a correlation matrix of the outcome variables and removing variables such that there are no correlations higher than 0.8 (or lower than -0.8) (variables removed shown in

Supplementary Table 4). Thus, these results are a subset of our main results, where the corrected P values are adjusted for the number of independent outcomes instead of the total number of outcomes. The number of results with $P < 0.05$ can decrease due to the removal of variables from the dataset, or increase because a smaller outcome set means that a smaller P value correction is needed. This approach removed 32 variables (none of which were in our validation set) to give an outcome set of size 140. This removed 2 of the outcomes with $p<0.05$ in the stage 1 results; the Attention/activity symptoms score and Apolipoprotein B. Thus after removing highly correlated variables, 12 of the 128 outcomes have a $P<0.05$ in the stage 1 tests. The Bonferroni approach found only 1 outcome with $P<0.05$ after adjustment for the 160 tests performed. This shows that the correlation method is less conservative than the Bonferroni approach (test of proportions $P = 0.0004$).

Supplementary Figure 1. A comparison of the instrumental variable estimates of the sensitivity analysis 52-SNP allele score and the original 32-SNP allele score for continuous outcomes

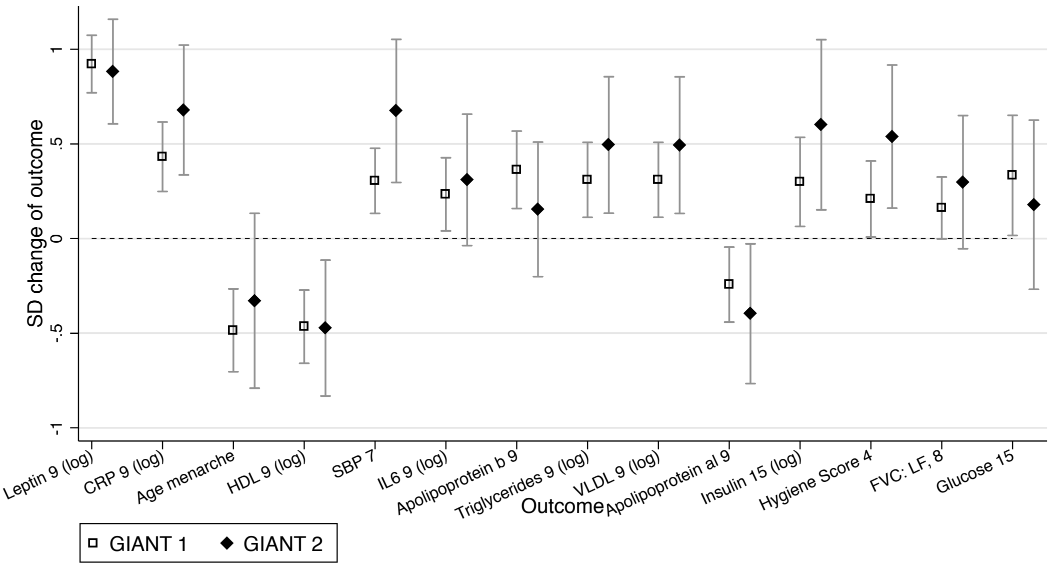

Supplementary Figure 2. A comparison of the instrumental variable estimates of the sensitivity analysis 52-SNP allele score and the original 32-SNP allele score for binary outcomes

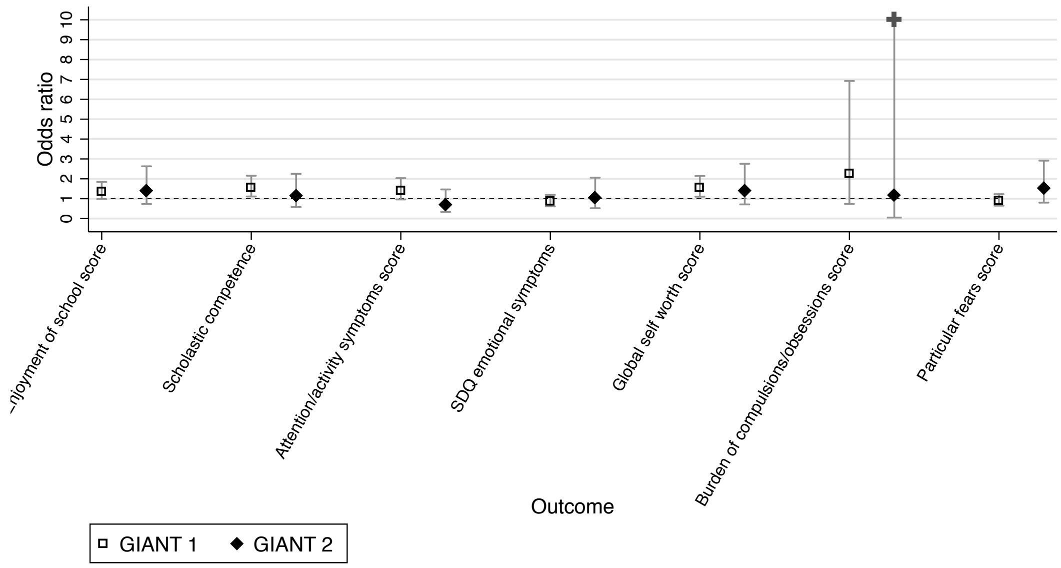

Supplementary Table 1. List of SNPs used to construct the BMI allele score

SNP	Imputation quality (r^2)	SNP	Imputation quality (r^2)
rs10150332	0.9963	rs2867125	0.9997
rs10767664	0.9965	rs2890652	0.9888
rs10938397	0.9881	rs29941	0.9999
rs10968576	0.9995	rs3810291	0.7652
rs11847697	0.9688	rs3817334	0.9984
rs12444979	0.9975	rs4771122	0.9313
rs13078807	0.9966	rs4836133	0.9429
rs13107325	0.9972	rs4929949	0.9671
rs1514175	0.9984	rs543874	0.9965
rs1555543	0.9960	rs571312	0.9995
rs1558902	0.9967	rs713586	0.9993
rs206936	0.9875	rs7138803	0.9980
rs2112347	0.9945	rs7359397	0.9988
rs2241423	0.9997	rs887912	0.9972
rs2287019	0.9991	rs9816226	0.9556
rs2815752	0.9964	rs987237	0.9994

BMI associated SNPs found in the largest GWAS to date (3).

Supplementary Table 2. ALSPAC Data files used to create the outcome dataset and the rules used to determine inclusion / exclusion of variables

Dataset	Age at measurement	Data file name	Variables chosen
Clinic – blood measures			
Focus age 9 - bloods	9 yrs 6 mnths	f_9_lipids	All variables included. Includes leptin and CRP variables used in validation set
Focus age 15 – Fasting bloods	15y 6m	fasting15_v9_nodups	Glucose and insulin for validation set
Clinic – other measures			
MacArthur CDI: saying and understanding scores	15m	CDI	All used, removed duplicates.
Focus age 7	7y 6m	f07_3d	We only included systolic blood pressure from this dataset, as one of our validation variables.
Focus age 8	8y 6m	f08_3b	This dataset contains 861 variables, and a set of derived scores. We kepts only variables representing the main concepts of this dataset, by including the scores. DANVA, WISC and lung function variables used for validation set.
Focus age 9	9y 6m	f09_3b	We used the main scores from this dataset - the "total raw accuracy score" and the "total raw comprehension score", included in our validation set.

Haemoglobin levels	7y 6m	haemoglobin_focus	This only contains haemoglobin, earliest timepoint used.
Coefficient of variation of total energy intake	10 years	cv_energy10y2a_aln	This only contains one energy measure – earliest timepoint used.
Questionnaires			
Age at menarche	n/a	age_at_menarche_mar12	This dataset only contains age at menarche, used in validation set.
Derived from strength and dificulties questionnaire	6 years 9m	sdq81mns_kq	All used, removed age and duplicates.
"{Girl/Boy}v Toddler questionnaire"	1year 6m	kd_4b	We used only the home score from this dataset, in our validation set.
3 year FFQ Nutrient Intake, derived from food frequencies in "My 3 Year old Boy/Girl"	3 yr 2 mnths	kgnut3yr_v3	All variables included.
"My Young 4 Year Old {Girl/Boy}"	4y 6m	kk_2c	We used only the scores from this dataset, and removed all duplicates of these.
"My {Daughter/Son} at School"	6y 9m	kq_2c	We used only the scores from this dataset, and removed all duplicates of these.
"My {Daughter/Sons} Wellbeing"	7y 7m	kr_1b	We used only the scores from this dataset, and removed all duplicates of these.
"Your {Daughter/Son} at 9"	9yr 7m	ku_r2b	We used only the scores from this dataset, and

			removed all duplicates of these.
Schools – “The Developing Child”, “Questionnaire for Class Teacher”, “Questionnaire for Head Teacher”	11yr 1m	sefg_1b	We used only the score variables.

For further information of ALSPAC variables See (4, 5) and the ALSPAC website: http://www.bristol.ac.uk/alspac/researchers/resources-available/

Supplementary Table 3. List of descriptions of outcome variables included in our dataset, from the ALSPAC cohort

Variable	Description	Timepoint	Validation set
chol_9	focus @ 9, cholesterol, mmol/l	9 yrs 6 mnths	
trig_9	focus @ 9, triglycerides, mmol/l	9 yrs 6 mnths	
vldl_9	focus @ 9, very low density lipoprotein, mmol/l	9 yrs 6 mnths	
ldl_9	focus @ 9, low density lipoprotein, mmol/l	9 yrs 6 mnths	
hdl_9	focus @ 9, high density lipoprotein, mmol/l	9 yrs 6 mnths	
apoai_9	focus @ 9, apolipoprotein al, mg/dl	9 yrs 6 mnths	
apob_9	focus @ 9, apolipoprotein b, mg/dl	9 yrs 6 mnths	
crp_9	focus @ 9, c-reactive protein,mg/l	9 yrs 6 mnths	YES
leptin_9	focus @ 9, leptin, ng/ml	9 yrs 6 mnths	YES
adiponectin_9	focus @ 9, adiponectin, ng/ml	9 yrs 6 mnths	
il6_9	focus @ 9, interleukin 6, pg/ml	9 yrs 6 mnths	
glucosem_15	glucose(mmol/l), 15 year fasting bloods	15 yrs 6 mnths	YES
insulini_15	insulin (iu/l), 15 year fasting bloods	15 yrs 6 mnths	YES
hb_f7	Haemoglobin at f@7	7 yrs 6 mnths	
AGE_MENARCHE_YEARS_comp	Age at menarche	n/a	YES
f7sa021	Mean BP systolic: samples F@7	7 yrs 6 mnths	YES
f9sn702	Total raw accuracy score: Story F@9	9 yrs 6 mnths	YES
f9sn703	Total raw comprehension score: Story F@9	9 yrs 6 mnths	YES
kc_und	DV: Number of words child understands (out of 134) at 15m	1 yr 3 mnths	
kc_says	DV: Number of words child can say (out of 134) at 15m	1 yr 3 mnths	
kgalcohol	daily alcohol intake (g) from ffq at 3years version 3	3 yr 2 mnths	
kgcalcium	daily calcium intake (mg) from ffq at 3years version 3	3 yr 2 mnths	
kgcarbohydrate	daily carbohydrate intake (g) from ffq at 3years version 3	3 yr 2 mnths	
kgcarotene	daily carotene intake (microgrammes) from ffq at 3years version 3	3 yr 2 mnths	
kgcholesterol	daily cholesterol intake (mg) from ffq at 3years version 3	3 yr 2 mnths	

kgenergy	daily energy intake (kj) from ffq at 3years version 3	3 yr 2 mnths
kgfat	daily fat intake (g) from ffq at 3years version 3	3 yr 2 mnths
kgn3	daily n-3 fatty acid intake (g) from fish only from ffq at 3years version 3	3 yr 2 mnths
kgdha	daily dha intake (g) from fish only from ffq at 3years version 3	3 yr 2 mnths
kgepa	daily epa intake (g) from fish only from ffq at 3years version 3	3 yr 2 mnths
kgfolate	daily folate intake (microgrammes) from ffq at 3years version 3	3 yr 2 mnths
kgiodine	daily iodine intake (microgrammes) from ffq at 3years version 3	3 yr 2 mnths
kgiron	daily iron intake (mg) from ffq at 3years version 3	3 yr 2 mnths
kgmg	daily magnesium intake (mg) from ffq at 3years version 3	3 yr 2 mnths
kgmono	daily monounsaturated fat intake (g) from ffq at 3years version 3	3 yr 2 mnths
kgnceq	daily niacin equivalents intake (mg) from ffq at 3years version 3	3 yr 2 mnths
kgnmesuga rs	daily nme sugars intake (g) from ffq at 3years version 3	3 yr 2 mnths
kgnsp	daily nsp intake (g) from ffq at 3years version 3	3 yr 2 mnths
kgphosphor us	daily phosphorus intake (mg) from ffq at 3years version 3	3 yr 2 mnths
kgpoly	daily polyunsaturated fat intake (g) from ffq at 3years version 3	3 yr 2 mnths
kgpotassiu m	daily potassium intake (mg) from ffq at 3years version 3	3 yr 2 mnths
kgprotein	daily protein intake (g) from ffq at 3years version 3	3 yr 2 mnths
kgretinol	daily retinol intake (microgrammes) from ffq at 3years version 3	3 yr 2 mnths
kgribo	daily riboflavin intake (mg) from ffq at 3years version 3	3 yr 2 mnths
kgsfa	daily saturated fat intake (g) from ffq at 3years version 3	3 yr 2 mnths
kgselenium	daily selenium intake (microgrammes) from ffq at 3years version 3	3 yr 2 mnths
kgsodium	daily sodium intake (mg) from ffq at 3years version 3	3 yr 2 mnths
kgstarch	daily starch intake (g) from ffq at 3years version 3	3 yr 2 mnths

kgsugar	daily sugar intake (g) from ffq at 3years version 3	3 yr 2 mnths	
kgthiamin	daily thiamin intake (mg) from ffq at 3years version 3	3 yr 2 mnths	
kgvitc	daily vitamin c intake (mg) from ffq at 3years version 3	3 yr 2 mnths	
kgvitb6	daily vitamin b6 intake (mg) from ffq at 3years version 3	3 yr 2 mnths	
kgvitb12	daily vitamin b12 intake (microgrammes) from ffq at 3years version 3	3 yr 2 mnths	
kgvitd	daily vitamin d intake (mg) from ffq at 3years version 3	3 yr 2 mnths	
kgvite	daily vitamin e intake (mg) from ffq at 3years version 3	3 yr 2 mnths	
kgzinc	daily zinc intake (mg) from ffq at 3years version 3	3 yr 2 mnths	
fd10cv_kcal	coefficient of variation for total energy intake (kcal), f10+	10 yrs	
kqpemotion	sdq emotional symptoms score (prorated)	6 yrs 9 mnths	
kqpconduct	sdq conduct problems score (prorated)	6 yrs 9 mnths	
kqphyper	sdq hyperactivity score (prorated)	6 yrs 9 mnths	
kqppeer	sdq peer problems score (prorated)	6 yrs 9 mnths	
kqpprosoc	sdq prosocial score (prorated)	6 yrs 9 mnths	
kqpebdtot	sdq total difficulties score (prorated)	6 yrs 9 mnths	
f8at065	Att Sky Search - Normative Score: F@8	8 yrs 6 mnths	
f8at146	Att Dual Task - Score: F@8	8 yrs 6 mnths	
f8at148	Att Dual Task - Normative Score: F@8	8 yrs 6 mnths	
f8at228	Att Opp Worlds Task - Normative score Same World: F@8	8 yrs 6 mnths	
f8at229	Att Opp Worlds Task - Normative score Opp World: F@8	8 yrs 6 mnths	
f8lc125	LoC - Locus of Control Score: F@8	8 yrs 6 mnths	
f8dv440	DANVA, All Faces - # Errors: F@8	8 yrs 6 mnths	YES
f8fs120	F&S Friends score: F@8	8 yrs 6 mnths	
f8aa150	Antisocial activities score: F@8	8 yrs 6 mnths	
f8gb041	Gender - CAI score: F@8	8 yrs 6 mnths	
f8bp026	Posting Behaviour - Irritability/Neg emotion Score: F@8	8 yrs 6 mnths	
f8bp036	Posting Behaviour - Impulsivity/Distractability Score: F@8	8 yrs 6 mnths	
f8bp046	Posting Behaviour - Approach Score: F@8	8 yrs 6 mnths	
f8bp056	Posting Behaviour - Sluggishness Score: F@8	8 yrs 6 mnths	
f8bp066	Posting Behaviour - Wariness Score: F@8	8 yrs 6 mnths	

f8se125	Self Esteem: Scholastic Competence Score: F@8	8 yrs 6 mnths	
f8se126	Self Esteem: Global Self Worth Score: F@8	8 yrs 6 mnths	
f8ws112	WISC - Total IQ: F@8	8 yrs 6 mnths	YES
f8ba026	Activities Behaviour - Irritability/Neg emotion Score: F@8	8 yrs 6 mnths	
f8ba036	Activities Behaviour - Impulsivity/Distractability Score: F@8	8 yrs 6 mnths	
f8ba046	Activities Behaviour - Approach Score: F@8	8 yrs 6 mnths	
f8ba056	Activities Behaviour - Sluggishness Score: F@8	8 yrs 6 mnths	
f8ba066	Activities Behaviour - Wariness Score: F@8	8 yrs 6 mnths	
f8sl040	S&L - WOLD comprehension - Raw Score: F@8	8 yrs 6 mnths	
f8lf110	SD score for FVC: LF, F@8	8 yrs 6 mnths	YES
se087b	DV: Activity symptoms score (prorated)	11 yrs 2 mnths (School year 6)	
se090b	DV: Attention symptoms score (prorated)	11 yrs 2 mnths (School year 6)	
se093b	DV: Attention/activity symptoms score (prorated)	11 yrs 2 mnths (School year 6)	
se098a	DV: Burden of attention/activity problems score	11 yrs 2 mnths (School year 6)	
se123b	DV: Awkward behaviours score (prorated)	11 yrs 2 mnths (School year 6)	
se126b	DV: Troublesome behaviours score (prorated)	11 yrs 2 mnths (School year 6)	
se129b	DV: Awkward/troublesome behaviours score (prorated)	11 yrs 2 mnths (School year 6)	
se134a	DV: Burden of awkward/troublesome behaviours score	11 yrs 2 mnths (School year 6)	
se161b	DV: SDQ prosocial score (prorated)	11 yrs 2 mnths (School year 6)	
se162b	DV: SDQ hyperactivity score (prorated)	11 yrs 2 mnths (School year 6)	
se163b	DV: SDQ emotional symptoms score (prorated)	11 yrs 2 mnths (School year 6)	
se164b	DV: SDQ conduct problems score (prorated)	11 yrs 2 mnths (School year 6)	
se165b	DV: SDQ peer problems score (prorated)	11 yrs 2 mnths (School year 6)	
se166b	DV: SDQ total difficulties score (prorated)	11 yrs 2 mnths (School year 6)	

sf573b	DV: CCEI anxiety subscale score (prorated)	11 yrs 2 mnths (School year 6)	
sf574b	DV: CCEI somatic subscale score (prorated)	11 yrs 2 mnths (School year 6)	
sf575b	DV: CCEI depression subscale score (prorated)	11 yrs 2 mnths (School year 6)	
sf576b	DV: CCEI total score (prorated)	11 yrs 2 mnths (School year 6)	
sf611b	DV: Bachman self esteem score (prorated)	11 yrs 2 mnths (School year 6)	
kd380a	HOME score	1 yr 6 mnths	YES
kk310	DV: Hygiene Score	4 yrs 6 mnths	
kk317	DV: Toilet Incontinence Score	4 yrs 6 mnths	
kk489	DV: CH Enjoyment of School Score	4 yrs 6 mnths	
kq316	DV: Sleep Worries Score	6 yrs 9 mnths	
kq378b	DV: Life events score since child's 5th birthday (prorated)	6 yrs 9 mnths	
kq425	DV: Locomotor Ability Score	6 yrs 9 mnths	
kq442	DV: Fine Motor Score	6 yrs 9 mnths	
kq462	DV: Cognitive Score	6 yrs 9 mnths	
kq475	DV: Playing & Sharing Score	6 yrs 9 mnths	
kq477	DV: Empathy Subscale Score	6 yrs 9 mnths	
kq486	DV: Ball Skills Score	6 yrs 9 mnths	
kq502	DV: Social Skills Score	6 yrs 9 mnths	
kq517	DV: Communication Score	6 yrs 9 mnths	
kq519	DV: Musical Subscale Score	6 yrs 9 mnths	
kq525	DV: Speech Intelligibility Score	6 yrs 9 mnths	
kq538	DV: Uncommunicative Score	6 yrs 9 mnths	
kq558	DV: Developmental Worries Score	6 yrs 9 mnths	
kq573	DV: Child Activity Score	6 yrs 9 mnths	
kq597	DV: Female Parenting Score	6 yrs 9 mnths	
kq622	DV: Male Parenting Score	6 yrs 9 mnths	
kq653	DV: Sibling Interaction Score	6 yrs 9 mnths	
kq680	DV: Feeding Difficulties Score	6 yrs 9 mnths	
kr213b	DV: Separation anxiety symptoms score (prorated)	7 yrs 7 mnths	
kr222a	DV: Burden of separation anxieties score	7 yrs 7 mnths	
kr236b	DV: Particular fears score (prorated)	7 yrs 7 mnths	
kr247a	DV: Burden of particular fears score	7 yrs 7 mnths	
kr259b	DV: Social fears score (prorated)	7 yrs 7 mnths	
kr275a	DV: Burden of social fears score	7 yrs 7 mnths	
kr300b	DV: Stress reactions score (prorated)	7 yrs 7 mnths	
kr309a	DV: Burden of stress reactions score	7 yrs 7 mnths	
kr332b	DV: Compulsions score (prorated)	7 yrs 7 mnths	
kr337b	DV: Compulsions/obsessions score (prorated)	7 yrs 7 mnths	

kr351a	DV: Burden of compulsions/obsessions score	7 yrs 7 mnths
kr367b	DV: General anxieties score (prorated)	7 yrs 7 mnths
kr379b	DV: General anxiety symptoms score (prorated)	7 yrs 7 mnths
kr387a	DV: Burden of general anxieties score	7 yrs 7 mnths
kr429a	DV: Burden of moods score	7 yrs 7 mnths
kr447b	DV: Activity symptoms score (prorated)	7 yrs 7 mnths
kr459b	DV: Attention symptoms score (prorated)	7 yrs 7 mnths
kr462b	DV: Attention/activity symptoms score (prorated)	7 yrs 7 mnths
kr468b	DV: Teacher complaints score (prorated)	7 yrs 7 mnths
kr478a	DV: Burden of attention/activity problems score	7 yrs 7 mnths
kr492b	DV: Awkward behaviours score (prorated)	7 yrs 7 mnths
kr501a	DV: Burden of awkward behaviours score	7 yrs 7 mnths
kr519b	DV: Troublesome behaviours score (prorated)	7 yrs 7 mnths
kr554b	DV: Skuse social cognition score (prorated)	7 yrs 7 mnths
ku503b	DV: CCC - Intelligibility and fluency score (prorated)	9 yrs 7 mnths
ku504b	DV: CCC - Syntax score (prorated)	9 yrs 7 mnths
ku505b	DV: CCC - Inappropriate initiation score (prorated)	9 yrs 7 mnths
ku506b	DV: CCC - Coherence score (prorated)	9 yrs 7 mnths
ku507b	DV: CCC - Stereotyped conversation score (prorated)	9 yrs 7 mnths
ku508b	DV: CCC - Use of conversational context score (prorated)	9 yrs 7 mnths
ku509b	DV: CCC - Conversational rapport score (prorated)	9 yrs 7 mnths
ku510b	DV: CCC - Pragmatic aspects of communication score (prorated)	9 yrs 7 mnths
ku673b	DV: SMFQ depression score (prorated)	9 yrs 7 mnths
ku705b	DV: SDQ - Prosocial score (prorated)	9 yrs 7 mnths
ku706b	DV: SDQ - Hyperactivity score (prorated)	9 yrs 7 mnths
ku707b	DV: SDQ - Emotional symptoms score (prorated)	9 yrs 7 mnths
ku708b	DV: SDQ - Conduct problems score (prorated)	9 yrs 7 mnths
ku709b	DV: SDQ - Peer problems score (prorated)	9 yrs 7 mnths

ku710b	DV: SDQ - Total difficulties score (prorated)	9 yrs 7 mnths
f8at060L	Selective attention	8 yrs 6 mnths
f8at061L	Motor score	8 yrs 6 mnths
f8at147L	Dual task decrement score	8 yrs 6 mnths

Abbreviations: yr, year; mnths, months; DV, derived variable; standard deviation; LDL, low density lipoprotein; WISC, Wechsler Intelligence Scale for Children; DANVA, Diagnostic Analysis of Nonverbal Accuracy test; IL6, interleukin 6; SBP, systolic blood pressure; HDL, high density lipoprotein; VLDL, very low density lipoprotein; SDQ, Strengths and Difficulties Questionnaires; CCC, Children's Communication Checklist; CCEI, Crown Crisp Experiental Index.

For further information of ALSPAC variables See (4, 5) and the ALSPAC website:
http://www.bristol.ac.uk/alspac/researchers/resources-available/

Variables included in the complete case dataset have prefix 'f8'

Supplementary Table 4. Ranking by association strength (P value) of the stage one tests: Outcome associations with BMI allele score for original and imputed datasets

Rank	Outcome variable (original data with variable N)	SD change of inverse normal transformed outcome for a 1 SD change of BMI allele score				SD change of inverse normal transformed imputed outcome for a 1 SD change of BMI allele score (N=8,121)			
		Sample size	SD change	95% CI	P value (adjusted P value) [2]	Imputed rank	SD change	95% CI	P value (adjusted P value) [2]
1	leptin_9 *	4,249	0.138	0.11, 0.17	<0.001	1	0.122	0.09, 0.15	<0.001
2	crp_9 *	4,250	0.083	0.05, 0.11	<0.001	3	0.072	0.04, 0.10	<0.001
3	AGE_MENARCHE_YE *	2,946	-0.083	-0.12, -0.05	<0.001	2	-0.087	-0.12, -0.06	<0.001
4	hdl_9	4,250	-0.067	-0.10, -0.04	<0.001 (0.002)	13	-0.040	-0.08, 0.00	0.028 (1)
5	f7sa021 *	6,013	0.049	0.02, 0.07	<0.001	4	0.045	0.02, 0.07	<0.001
6	il6_9	4,240	0.053	0.02, 0.08	0.001 (0.091)	8	0.043	0.01, 0.08	0.011 (1)
7	kk489	5,807	0.041	0.02, 0.07	0.002 (0.255)	5	0.042	0.02, 0.07	0.001 (0.188)
8	f8se125	5,222	0.042	0.02, 0.07	0.002 (0.323)	6	0.039	0.01, 0.07	0.005 (0.787)
9	apob_9	4,250	0.043	0.01, 0.07	0.005 (0.788)	37	0.021	-0.01, 0.05	0.160 (1)
10	trig_9	4,250	0.042	0.01, 0.07	0.006 (0.970)	33	0.022	-0.01, 0.05	0.152 (1)
11	vldl_9	4,250	0.042	0.01, 0.07	0.006 (1)	34	0.022	-0.01, 0.05	0.157 (1)
12	apoai_9	4,250	-0.038	-0.07, -0.01	0.012 (1)	42	-0.022	-0.05, 0.01	0.198 (1)
13	insulini_15 *	2,859	0.047	0.01, 0.08	0.012	9	0.045	0.01, 0.08	0.016
14	se093b	4,541	0.037	0.01, 0.07	0.013 (1)	18	0.030	0.00, 0.06	0.041 (1)
15	kqpemotion	5,748	-0.030	-0.06, 0.00	0.022 (1)	14	-0.030	-0.06, 0.00	0.030 (1)
16	kk310	6,231	0.028	0.00, 0.05	0.024 (1)	20	0.027	0.00, 0.05	0.048 (1)
17	f8se126	5,214	0.031	0.00, 0.06	0.025 (1)	24	0.026	0.00, 0.05	0.076 (1)
18	f8lf110 *	5,276	0.030	0.00, 0.06	0.030 (1)	7	0.033	0.01, 0.06	0.008 (1)

19	kr351a	5,684	0.028	0.00, 0.05	0.031 (1)	15	0.026	0.00, 0.05	0.036 (1)
20	kr236b	5,734	-0.028	-0.05, 0.00	0.033 (1)	11	-0.030	-0.06, 0.00	0.019 (1)
21	glucosem_15 *	2,862	0.041	0.00, 0.08	0.038	16	0.041	0.00, 0.08	0.037
22	se129b	4,545	0.028	0.00, 0.06	0.051 (1)	10	0.030	0.00, 0.05	0.019 (1)
23	se123b	4,545	0.029	0.00, 0.06	0.052 (1)	12	0.030	0.00, 0.05	0.022 (1)
24	kr367b	5,703	-0.025	-0.05, 0.00	0.054 (1)	17	-0.027	-0.05, 0.00	0.040 (1)
25	se162b	4,546	0.028	0.00, 0.06	0.059 (1)	38	0.021	-0.01, 0.05	0.163 (1)
26	kq538	5,798	-0.025	-0.05, 0.00	0.062 (1)	22	-0.024	-0.05, 0.00	0.068 (1)
27	kr247a	5,299	-0.025	-0.05, 0.00	0.065 (1)	21	-0.025	-0.05, 0.00	0.061 (1)
28	kr379b	5,674	-0.023	-0.05, 0.00	0.072 (1)	19	-0.027	-0.05, 0.00	0.045 (1)
29	kq475	5,769	-0.023	-0.05, 0.00	0.078 (1)	23	-0.023	-0.05, 0.00	0.072 (1)
30	kr259b	5,692	-0.023	-0.05, 0.00	0.085 (1)	25	-0.023	-0.05, 0.00	0.076 (1)
31	ku709b	5,746	-0.022	-0.05, 0.00	0.089 (1)	30	-0.021	-0.05, 0.01	0.124 (1)
32	kgnsp	6,301	0.022	0.00, 0.05	0.090 (1)	29	0.019	0.00, 0.04	0.114 (1)
33	kgcarotene	6,301	0.021	0.00, 0.05	0.095 (1)	35	0.018	-0.01, 0.04	0.158 (1)
34	se090b	4,541	0.024	-0.01, 0.05	0.105 (1)	28	0.021	0.00, 0.05	0.107 (1)
35	adiponectin_9	4,247	-0.024	-0.05, 0.01	0.126 (1)	59	-0.016	-0.05, 0.02	0.326 (1)
36	se134a	4,514	0.022	-0.01, 0.05	0.129 (1)	41	0.021	-0.01, 0.05	0.196 (1)
37	ku705b	5,755	0.020	-0.01, 0.05	0.132 (1)	40	0.017	-0.01, 0.04	0.191 (1)
38	se165b	4,546	0.023	-0.01, 0.05	0.132 (1)	27	0.026	-0.01, 0.06	0.105 (1)
39	kq573	5,798	0.019	-0.01, 0.04	0.137 (1)	26	0.022	0.00, 0.05	0.097 (1)
40	kq622	5,635	0.020	-0.01, 0.05	0.141 (1)	32	0.021	-0.01, 0.05	0.129 (1)
41	f8at229	5,416	0.020	-0.01, 0.05	0.142 (1)	45	0.016	-0.01, 0.04	0.221 (1)
42	ldl_9	4,250	0.022	-0.01, 0.05	0.152 (1)	94	0.009	-0.02, 0.04	0.541 (1)
43	f8ba066	5,572	-0.019	-0.04, 0.01	0.158 (1)	36	-0.018	-0.04, 0.01	0.159 (1)
44	f8ba026	5,581	0.018	-0.01, 0.04	0.177 (1)	31	0.020	-0.01, 0.05	0.128 (1)
45	ku710b	5,732	-0.017	-0.04, 0.01	0.179 (1)	56	-0.014	-0.04, 0.01	0.298 (1)
46	kgvitb12	6,301	-0.017	-0.04, 0.01	0.181 (1)	43	-0.016	-0.04, 0.01	0.198 (1)
47	f8aa150	5,367	-0.018	-0.04, 0.01	0.184 (1)	51	-0.018	-0.05, 0.01	0.249 (1)
48	f8ba056	5,572	-0.018	-0.04, 0.01	0.184 (1)	52	-0.015	-0.04, 0.01	0.259 (1)
49	ku509b	5,693	0.017	-0.01, 0.04	0.196 (1)	54	0.015	-0.01, 0.04	0.294 (1)

50	f8bp046	5,559	0.017	-0.01, 0.04	0.199 (1)	84	0.009	-0.02, 0.04	0.493 (1)
51	sf574b	4,296	0.019	-0.01, 0.05	0.202 (1)	47	0.018	-0.01, 0.05	0.232 (1)
52	kq519	5,770	0.016	-0.01, 0.04	0.211 (1)	60	0.013	-0.01, 0.04	0.327 (1)
53	ku503b	5,793	0.016	-0.01, 0.04	0.219 (1)	63	0.012	-0.01, 0.04	0.335 (1)
54	kqpebdtot	5,724	-0.015	-0.04, 0.01	0.235 (1)	46	-0.015	-0.04, 0.01	0.227 (1)
55	se164b	4,545	0.017	-0.01, 0.05	0.241 (1)	70	0.013	-0.02, 0.04	0.386 (1)
56	kq597	5,748	0.015	-0.01, 0.04	0.258 (1)	44	0.016	-0.01, 0.04	0.212 (1)
57	kgretinol	6,301	-0.014	-0.04, 0.01	0.279 (1)	48	-0.014	-0.04, 0.01	0.235 (1)
58	ku707b	5,736	-0.014	-0.04, 0.01	0.290 (1)	57	-0.013	-0.04, 0.01	0.314 (1)
59	kr519b	5,664	0.013	-0.01, 0.04	0.308 (1)	83	0.010	-0.02, 0.04	0.473 (1)
60	kq525	5,765	-0.013	-0.04, 0.01	0.312 (1)	55	-0.014	-0.04, 0.01	0.296 (1)
61	f8sl040	5,560	-0.014	-0.04, 0.01	0.313 (1)	39	-0.017	-0.04, 0.01	0.186 (1)
62	se166b	4,546	0.015	-0.01, 0.04	0.327 (1)	74	0.012	-0.02, 0.04	0.403 (1)
63	sf611b	4,302	-0.014	-0.04, 0.01	0.327 (1)	71	-0.013	-0.04, 0.02	0.387 (1)
64	kgvitd	6,301	-0.013	-0.04, 0.01	0.328 (1)	62	-0.012	-0.04, 0.01	0.331 (1)
65	kq680	5,798	-0.013	-0.04, 0.01	0.336 (1)	49	-0.016	-0.04, 0.01	0.236 (1)
66	kgmg	6,301	0.012	-0.01, 0.04	0.345 (1)	61	0.011	-0.01, 0.03	0.329 (1)
67	kr492b	5,661	0.012	-0.01, 0.04	0.353 (1)	80	0.010	-0.02, 0.04	0.456 (1)
68	ku504b	5,775	-0.012	-0.04, 0.01	0.355 (1)	50	-0.016	-0.04, 0.01	0.240 (1)
69	f8dv440 *	5,108	-0.013	-0.04, 0.01	0.355	67	-0.012	-0.04, 0.01	0.361
70	kr447b	5,680	0.011	-0.01, 0.04	0.383 (1)	106	0.006	-0.02, 0.03	0.629 (1)
71	kr309a	5,665	-0.011	-0.04, 0.01	0.413 (1)	77	-0.011	-0.04, 0.02	0.430 (1)
72	f8ba036	5,567	-0.011	-0.04, 0.01	0.415 (1)	115	-0.006	-0.04, 0.02	0.690 (1)
73	f8at065	5,483	0.011	-0.02, 0.04	0.422 (1)	58	0.014	-0.01, 0.04	0.321 (1)
74	kgcalcium	6,301	-0.010	-0.03, 0.01	0.423 (1)	82	-0.009	-0.03, 0.01	0.458 (1)
75	kgiron	6,301	0.010	-0.01, 0.03	0.426 (1)	66	0.011	-0.01, 0.03	0.356 (1)
76	ku706b	5,756	-0.010	-0.04, 0.02	0.433 (1)	91	-0.008	-0.03, 0.02	0.537 (1)
77	kgvite	6,301	-0.010	-0.03, 0.02	0.442 (1)	81	-0.009	-0.03, 0.01	0.457 (1)
78	f8at061	5,427	-0.010	-0.04, 0.02	0.444 (1)	118	-0.005	-0.03, 0.02	0.694 (1)
79	kr429a	5,653	0.010	-0.02, 0.04	0.448 (1)	87	0.009	-0.02, 0.04	0.513 (1)
80	kgfolate	6,301	0.009	-0.02, 0.03	0.458 (1)	75	0.010	-0.01, 0.03	0.412 (1)

81	f8bp026	5,573	-0.010	-0.04, 0.02	0.461 (1)	116	-0.005	-0.03, 0.02	0.692 (1)
82	kqphyper	5,748	-0.010	-0.04, 0.02	0.464 (1)	73	-0.010	-0.03, 0.01	0.401 (1)
83	se161b	4,546	-0.011	-0.04, 0.02	0.472 (1)	72	-0.012	-0.04, 0.02	0.399 (1)
84	kc_und	6,853	-0.009	-0.03, 0.02	0.476 (1)	69	-0.011	-0.03, 0.01	0.372 (1)
85	chol_9	4,250	0.011	-0.02, 0.04	0.478 (1)	127	0.005	-0.03, 0.04	0.750 (1)
86	kgcholesterol	6,301	-0.009	-0.03, 0.02	0.488 (1)	85	-0.008	-0.03, 0.01	0.496 (1)
87	kr462b	5,689	0.009	-0.02, 0.03	0.488 (1)	103	0.006	-0.02, 0.03	0.618 (1)
88	ku505b	5,770	-0.009	-0.03, 0.02	0.491 (1)	53	-0.015	-0.04, 0.01	0.284 (1)
89	kgiodine	6,301	0.008	-0.02, 0.03	0.499 (1)	68	0.011	-0.01, 0.03	0.371 (1)
90	kgpoly	6,301	-0.008	-0.03, 0.02	0.507 (1)	88	-0.008	-0.03, 0.02	0.517 (1)
91	kq477	5,769	-0.008	-0.03, 0.02	0.527 (1)	79	-0.009	-0.03, 0.01	0.449 (1)
92	se087b	4,537	0.009	-0.02, 0.04	0.527 (1)	135	0.004	-0.02, 0.03	0.781 (1)
93	kgstarch	6,301	0.008	-0.02, 0.03	0.528 (1)	89	0.008	-0.02, 0.03	0.534 (1)
94	kq502	5,765	0.008	-0.02, 0.03	0.535 (1)	105	0.006	-0.02, 0.03	0.622 (1)
95	f8bp036	5,559	-0.008	-0.03, 0.02	0.544 (1)	132	-0.004	-0.03, 0.02	0.779 (1)
96	sf576b	4,318	0.009	-0.02, 0.04	0.546 (1)	122	0.006	-0.02, 0.04	0.707 (1)
97	kgdha	6,301	0.008	-0.02, 0.03	0.547 (1)	102	0.006	-0.02, 0.03	0.616 (1)
98	kgselenium	6,301	0.008	-0.02, 0.03	0.558 (1)	96	0.007	-0.02, 0.03	0.567 (1)
99	hb_f7	4,761	0.008	-0.02, 0.04	0.560 (1)	121	0.006	-0.02, 0.03	0.705 (1)
100	kq425	5,777	-0.007	-0.03, 0.02	0.576 (1)	76	-0.010	-0.03, 0.01	0.417 (1)
101	kd380a *	6,885	0.007	-0.02, 0.03	0.581	99	0.006	-0.02, 0.03	0.598
102	f8ba046	5,565	0.007	-0.02, 0.03	0.582 (1)	97	0.007	-0.02, 0.03	0.589 (1)
103	kgn3	6,301	0.007	-0.02, 0.03	0.588 (1)	112	0.005	-0.02, 0.03	0.679 (1)
104	kqppeer	5,752	-0.007	-0.03, 0.02	0.592 (1)	90	-0.008	-0.03, 0.02	0.535 (1)
105	ku708b	5,751	-0.007	-0.03, 0.02	0.593 (1)	158	-0.001	-0.03, 0.03	0.921 (1)
106	kqpconduct	5,755	0.007	-0.02, 0.03	0.595 (1)	104	0.006	-0.02, 0.03	0.621 (1)
107	kr332b	5,651	-0.007	-0.03, 0.02	0.595 (1)	78	-0.010	-0.04, 0.02	0.432 (1)
108	kgepa	6,301	0.007	-0.02, 0.03	0.595 (1)	111	0.005	-0.02, 0.03	0.675 (1)
109	f8gb041	5,301	-0.007	-0.03, 0.02	0.598 (1)	109	-0.006	-0.03, 0.02	0.664 (1)
110	kc_says	6,853	-0.006	-0.03, 0.02	0.610 (1)	92	-0.007	-0.03, 0.02	0.538 (1)
111	kq558	5,798	-0.007	-0.03, 0.02	0.614 (1)	120	-0.005	-0.03, 0.02	0.699 (1)

112	kr222a	5,683	-0.007	-0.03, 0.02	0.625 (1)	65	-0.013	-0.04, 0.01	0.345 (1)
113	f8at228	5,420	0.007	-0.02, 0.03	0.632 (1)	124	0.005	-0.02, 0.03	0.735 (1)
114	fd10cv_kcal	4,922	-0.007	-0.03, 0.02	0.643 (1)	64	-0.014	-0.04, 0.01	0.339 (1)
115	kr554b	5,666	0.006	-0.02, 0.03	0.648 (1)	152	0.002	-0.02, 0.03	0.879 (1)
116	kr478a	5,476	-0.006	-0.03, 0.02	0.659 (1)	163	0.001	-0.03, 0.03	0.943 (1)
117	f9sn702 *	5,286	-0.006	-0.03, 0.02	0.659	160	-0.001	-0.03, 0.02	0.930
118	se098a	4,420	0.006	-0.02, 0.04	0.662 (1)	128	0.004	-0.02, 0.03	0.751 (1)
119	kgfat	6,301	-0.005	-0.03, 0.02	0.679 (1)	110	-0.005	-0.03, 0.02	0.667 (1)
120	f8fs120	5,365	-0.005	-0.03, 0.02	0.687 (1)	129	-0.004	-0.03, 0.02	0.763 (1)
121	ku506b	5,775	0.005	-0.02, 0.03	0.688 (1)	153	0.002	-0.02, 0.03	0.883 (1)
122	ku508b	5,695	0.005	-0.02, 0.03	0.693 (1)	145	0.003	-0.02, 0.03	0.835 (1)
123	kr459b	5,673	0.005	-0.02, 0.03	0.695 (1)	101	0.007	-0.02, 0.03	0.616 (1)
124	kr213b	5,631	-0.005	-0.03, 0.02	0.704 (1)	93	-0.008	-0.03, 0.02	0.539 (1)
125	kgsfa	6,301	-0.005	-0.03, 0.02	0.705 (1)	117	-0.005	-0.03, 0.02	0.693 (1)
126	ku673b	5,749	-0.005	-0.03, 0.02	0.713 (1)	138	-0.003	-0.03, 0.02	0.800 (1)
127	kq442	5,761	-0.005	-0.03, 0.02	0.721 (1)	95	-0.007	-0.03, 0.02	0.552 (1)
128	kr300b	5,708	0.005	-0.02, 0.03	0.722 (1)	125	0.004	-0.02, 0.03	0.748 (1)
129	kq486	5,758	0.005	-0.02, 0.03	0.725 (1)	150	0.002	-0.02, 0.03	0.858 (1)
130	kgcarbohydrate	6,301	0.004	-0.02, 0.03	0.745 (1)	114	0.005	-0.02, 0.03	0.685 (1)
131	kgribo	6,301	-0.004	-0.03, 0.02	0.750 (1)	154	-0.002	-0.02, 0.02	0.888 (1)
132	kgnmesugars	6,301	0.004	-0.02, 0.03	0.751 (1)	100	0.006	-0.02, 0.03	0.599 (1)
133	kgprotein	6,301	-0.004	-0.03, 0.02	0.754 (1)	131	-0.003	-0.03, 0.02	0.769 (1)
134	f8at147	5,312	-0.004	-0.03, 0.02	0.765 (1)	161	-0.001	-0.03, 0.02	0.939 (1)
135	kr275a	5,718	-0.004	-0.03, 0.02	0.767 (1)	146	-0.003	-0.03, 0.02	0.836 (1)
136	f8ws112 *	5,516	-0.004	-0.03, 0.02	0.769	86	-0.008	-0.03, 0.02	0.513
137	kq316	5,780	0.004	-0.02, 0.03	0.782 (1)	123	0.005	-0.02, 0.03	0.717 (1)
138	f8at060	5,447	-0.004	-0.03, 0.02	0.791 (1)	139	-0.003	-0.03, 0.02	0.801 (1)
139	kgphosphorus	6,301	-0.003	-0.03, 0.02	0.798 (1)	130	-0.003	-0.03, 0.02	0.768 (1)
140	kgzinc	6,301	-0.003	-0.03, 0.02	0.798 (1)	136	-0.003	-0.03, 0.02	0.789 (1)
141	f9sn703 *	5,286	0.003	-0.02, 0.03	0.800	98	0.007	-0.02, 0.03	0.589
142	ku510b	5,668	-0.003	-0.03, 0.02	0.805 (1)	113	-0.006	-0.03, 0.02	0.683 (1)

143	sf575b	4,318	0.004	-0.03, 0.03	0.817 (1)	164	0.001	-0.03, 0.03	0.945 (1)
144	f8lc125	4,793	-0.003	-0.03, 0.03	0.824 (1)	134	-0.004	-0.03, 0.02	0.781 (1)
145	se163b	4,546	-0.003	-0.03, 0.03	0.830 (1)	157	-0.002	-0.03, 0.03	0.917 (1)
146	kq378b	5,775	0.003	-0.02, 0.03	0.833 (1)	147	0.003	-0.02, 0.03	0.843 (1)
147	kgmono	6,301	-0.002	-0.03, 0.02	0.842 (1)	155	-0.001	-0.02, 0.02	0.896 (1)
148	f8bp066	5,566	-0.003	-0.03, 0.02	0.849 (1)	165	0.001	-0.03, 0.03	0.949 (1)
149	kqpprosoc	5,754	-0.002	-0.03, 0.02	0.858 (1)	143	-0.003	-0.03, 0.02	0.820 (1)
150	kr387a	5,673	0.002	-0.02, 0.03	0.859 (1)	142	-0.003	-0.03, 0.02	0.820 (1)
151	kr501a	5,700	0.002	-0.02, 0.03	0.879 (1)	149	0.002	-0.02, 0.03	0.858 (1)
152	kgsodium	6,301	0.002	-0.02, 0.03	0.883 (1)	151	0.002	-0.02, 0.02	0.873 (1)
153	kr468b	5,688	0.002	-0.02, 0.03	0.886 (1)	156	0.002	-0.03, 0.03	0.904 (1)
154	f8at146	5,340	-0.002	-0.03, 0.02	0.889 (1)	172	0.000	-0.03, 0.03	0.999 (1)
155	kgvitb6	6,301	-0.002	-0.03, 0.02	0.896 (1)	140	-0.003	-0.03, 0.02	0.802 (1)
156	kk317	6,209	0.002	-0.02, 0.03	0.898 (1)	137	0.003	-0.02, 0.03	0.795 (1)
157	kq653	5,216	0.002	-0.03, 0.03	0.902 (1)	108	0.006	-0.02, 0.03	0.661 (1)
158	kgalcohol	6,301	-0.002	-0.03, 0.02	0.906 (1)	148	-0.002	-0.03, 0.02	0.854 (1)
159	kq517	5,771	-0.001	-0.03, 0.02	0.908 (1)	162	-0.001	-0.03, 0.03	0.941 (1)
160	sf573b	4,321	-0.002	-0.03, 0.03	0.912 (1)	119	-0.006	-0.04, 0.02	0.697 (1)
161	kgpotassium	6,301	0.001	-0.02, 0.03	0.917 (1)	144	0.002	-0.02, 0.03	0.830 (1)
162	kq462	5,774	0.001	-0.02, 0.03	0.919 (1)	126	0.004	-0.02, 0.03	0.749 (1)
163	kr337b	5,651	-0.001	-0.03, 0.02	0.922 (1)	133	-0.004	-0.03, 0.02	0.779 (1)
164	kgsugar	6,301	-0.001	-0.03, 0.02	0.939 (1)	169	0.000	-0.02, 0.02	0.983 (1)
165	kgvitc	6,301	0.001	-0.02, 0.03	0.945 (1)	159	-0.001	-0.03, 0.02	0.924 (1)
166	kgthiamin	6,301	0.001	-0.02, 0.03	0.951 (1)	171	0.000	-0.02, 0.02	0.993 (1)
167	f8bp056	5,567	0.001	-0.03, 0.03	0.968 (1)	107	0.006	-0.02, 0.03	0.631 (1)
168	f8at148	5,315	0.001	-0.03, 0.03	0.968 (1)	170	0.000	-0.02, 0.02	0.988 (1)
169	kgnceq	6,301	0.000	-0.02, 0.03	0.971 (1)	166	0.000	-0.02, 0.02	0.968 (1)
170	se126b	4,545	0.000	-0.03, 0.03	0.975 (1)	168	0.001	-0.03, 0.03	0.970 (1)
171	kgenergy	6,301	0.000	-0.02, 0.02	0.990 (1)	167	0.000	-0.02, 0.02	0.969 (1)
172	ku507b	5,751	0.000	-0.03, 0.03	1.000 (1)	141	-0.004	-0.03, 0.03	0.812 (1)

Full names of variables are given in Supplementary Table 3.

All outcomes are transformed to normal distributions using a rank-based inverse normal transformation. Exposure and outcome variables are standardised. Outcome as dependent variable, BMI allele score as independent variable.

[1] Using Stata `IVregress` command and `robust` option. BMI allele score is the instrumental variable for log BMI age 8. First stage predicting log bmi at age 8. The second stage performs an unadjusted association of these log bmi age 8 predictions with the outcome.

[2]Adjusted P values are adjusted for the 160 tests performed using the Bonferroni correction: $p_{corrected} = p_{original} \cdot 160$. Adjusted P values greater than 1 are rounded to 1.

* Variables in validation set

Supplementary Table 5. Variable removed from the outcome dataset because they are highly correlated with another outcome, and the outcome they are correlated with

Outcome kept and representing this subset of dependent outcomes	Outcome removed from dataset
f8at060	f8at065
f8at146	f8at147
f8at147	f8at148
se090b	se093b
sf573b	sf576b
chol_9	ldl_9
trig_9	vldl_9
ldl_9	apob_9
hdl_9	apoai_9
kgcalcium	kgiodine kgphosphorus kgpotassium kgribo kgzinc
kgcarbohydrate	kgenergy kgiron kgnmesugars kgsodium kgstarch kgsugar
kgenergy	kgfat kgmg kgmono kgnceq kgprotein kgsfa
kgn3	kgdha kgepa
kgfolate	kgthiamin kgvitb6
kgiron	kgnsp
kgpoly	kgvite

Full names of variables are given in Supplementary Table 2

[1] Variables with P < 0.05 for the results of the directed tests shown in Table 3

Supplementary Table 6. List of the variables associated with the BMI allele score, with abbreviated and full variable names

Abbreviated variable name	Variable
Leptin, 9	leptin_9
CRP, 9	crp_9
Age menarche	AGE_MENARCHE_YE
HDL, 9	hdl_9
SBP, 7	f7sa021
IL6, 9	il6_9
Enjoyment of School Score, 4	kk489
Self Esteem: Scholastic Competence, 8	f8se125
Apolipoprotein B, 9	apob_9
Triglycerides, 9	trig_9
VLDL age 9	vldl_9

Apolipoprotein al, 9	apoai_9
Insulin, 15	insulini_15
Attention/activity symptoms score, 11	se093b
SDQ emotional symptoms score, 6	kqpemotion
Hygiene Score, 4	kk310
Self Esteem: Global Self Worth Score, 8	f8se126
FVC: LF, 8	f8lf110
Burden of compulsions/obsessions score, 7	kr351a
Particular fears score, 7	kr236b
Glucose, 15	glucosem_15

Abbreviations: BMI, body mass index; CI, confidence interval; SD, standard deviation; IV, instrumental variable; CRP, c-reactive protein; LDL, low density lipoprotein; IL6, interleukin 6; SBP, systolic blood pressure; HDL, high density lipoprotein; VLDL, very low density lipoprotein; SDQ, Strengths and Difficulties Questionnaires; LF, lung function; FVC, forced vital capacity;

Supplementary Table 7. Data transformations of outcome variables used in stage 2 analysis

Outcome variable	Data transformation	
Leptin 9	Logarithm	
CRP, 9	Logarithm	
HDL, 9	Logarithm	
IL6, 9	Logarithm	
VLDL, 9	Logarithm	
Triglycerides, 9	Logarithm	
Insulin, 15	Logarithm	
Enjoyment of School Score, 4	Binary	<19, ≥19
Self Esteem: Scholastic Competence, 8	Binary	<18, ≥18
Attention/activity symptoms score, 11	Binary	0, >0
SDQ emotional symptoms score, 6	Binary	0, >0
Self Esteem: Global Self Worth Score, 8	Binary	<20, ≥20
Burden of compulsions/obsessions score, 7	Binary	0, >0
Particular fears score, 7	Binary	<4, ≥4

Logarithm tranformations use base 10.

Supplementary Table 8: List of the 52 SNPs used to generate the sensitivity analysis 52-SNP allele score

rs12286929	rs17001654	rs9641123
rs7903146	rs11191560	rs7164727
rs10132280	rs1528435	rs492400
rs17094222	rs1000940	rs2080454
rs7599312	rs2033529	rs7239883
rs2365389	rs9400239	rs2836754
rs12885454	rs10733682	rs9914578
rs16851483	rs11688816	rs9374842
rs1167827	rs11057405	rs4787491
rs758747	rs11727676	rs1441264
rs1928295	rs3849570	rs17203016
rs9925964	rs6477694	rs16907751
rs11126666	rs7899106	rs13201877
rs2650492	rs2176598	rs9540493
rs6804842	rs2245368	rs1460676
rs4740619	rs17724992	rs6465468
rs13191362	rs7243357	
rs3736485	rs2033732	

Source of SNPs *(6)*

REFERENCES

1. White IR, Royston P, Wood AM. Multiple imputation using chained equations: Issues and guidance for practice. *Statistics in medicine* 2011;30(4):377-99.
2. Royston P. Multiple imputation of missing values: update. *Stata J* 2005;5(2):188-201.
3. Speliotes EK, Willer CJ, Berndt SI, et al. Association analyses of 249,796 individuals reveal 18 new loci associated with body mass index. *Nat Genet* 2010;42(11):937-48.
4. Fraser A, Macdonald-Wallis C, Tilling K, et al. Cohort Profile: The Avon Longitudinal Study of Parents and Children: ALSPAC mothers cohort. *International journal of epidemiology* 2012;In press.
5. Golding J, Pembrey M, Jones R. ALSPAC--the Avon Longitudinal Study of Parents and Children. I. Study methodology. *Paediatr Perinat Epidemiol* 2001;15(1):74-87.
6. Locke AE, Kahali B, Berndt SI, et al. Genetic studies of body mass index yield new insights for obesity biology. *Nature* 2015;518(7538):197-206.
